# Supplementary material for: Ternary nickel–tungsten–copper alloy rivals platinum for catalyzing alkaline hydrogen oxidation
Source: Nat Commun. 2021 May 11;12:2686. doi: 10.1038/s41467-021-22996-2 (PMC8113563; doi:10.1038/s41467-021-22996-2)
Supplement: Supplementary file 1 — Supplementary Information [file 41467_2021_22996_MOESM1_ESM.pdf]

Supplementary Information for

Ternary nickel-tungsten-copper alloy rivals platinum for catalyzing alkaline hydrogen oxidation

Shuai Qin[1†], Yu Duan[1†], Xiao-Long Zhang[1†], Li-Rong Zheng[2], Fei-Yue Gao[1], Peng-Peng Yang[1], Zhuang-Zhuang Niu[1], Ren Liu[3], Yu Yang[1], Xu-Sheng Zheng[4], Jun-Fa Zhu[4], Min-Rui Gao[1⋆]

Supplementary Figures

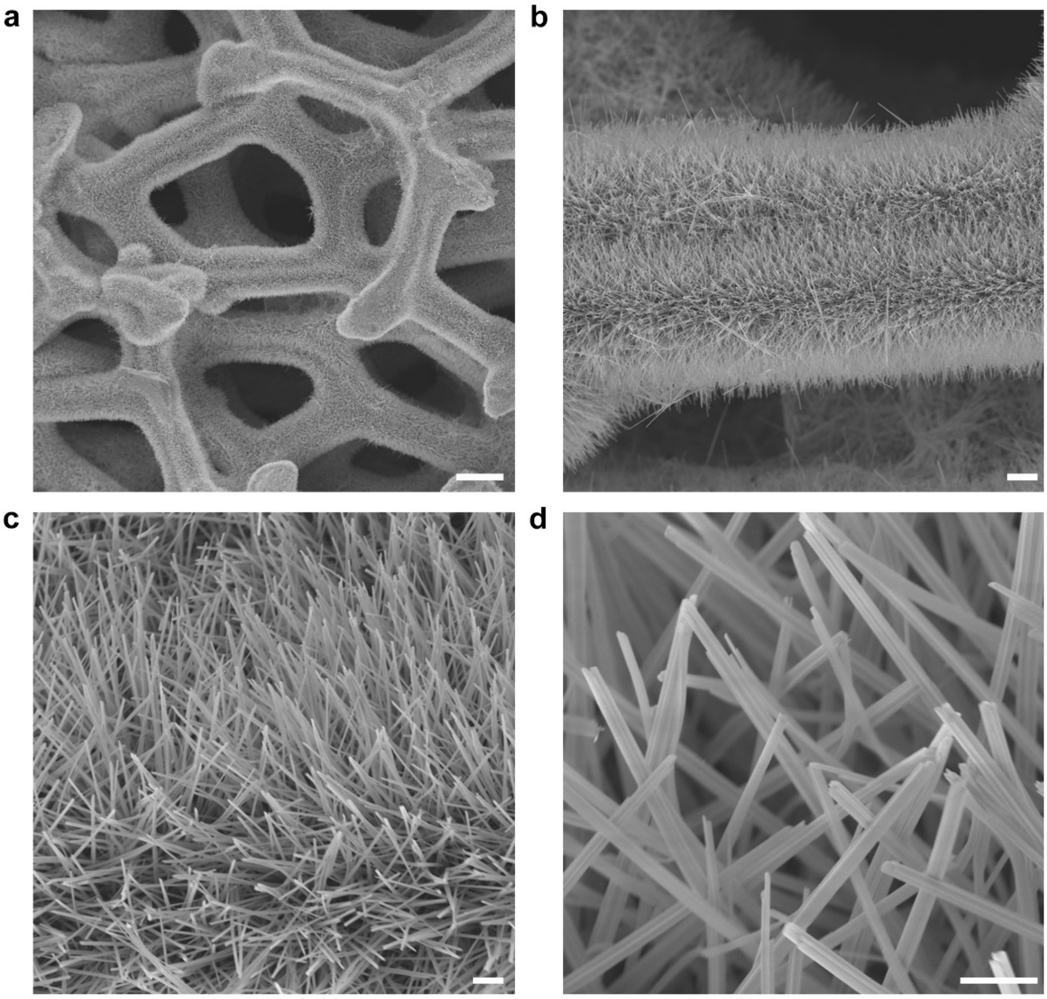

Supplementary Figure 1. SEM images of $Cu(OH)_2$ NWs grown on Cu foam via anodization. Scale bars, 100 μm (**a**), 10 μm (**b**), 2 μm (**c**) and 1 μm (**d**).

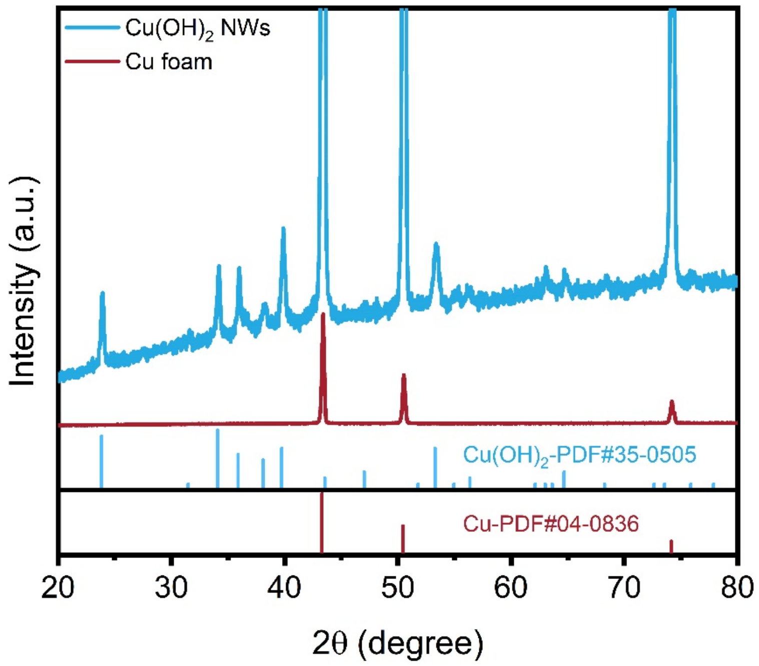

Supplementary Figure 2. XRD patterns of $Cu(OH)_2$ NWs and pure Cu foam. The inserted lines are standard XRD patterns for $Cu(OH)_2$ (*JCPDS* 35-0505) and Cu (*JCPDS* 04-0836), respectively.

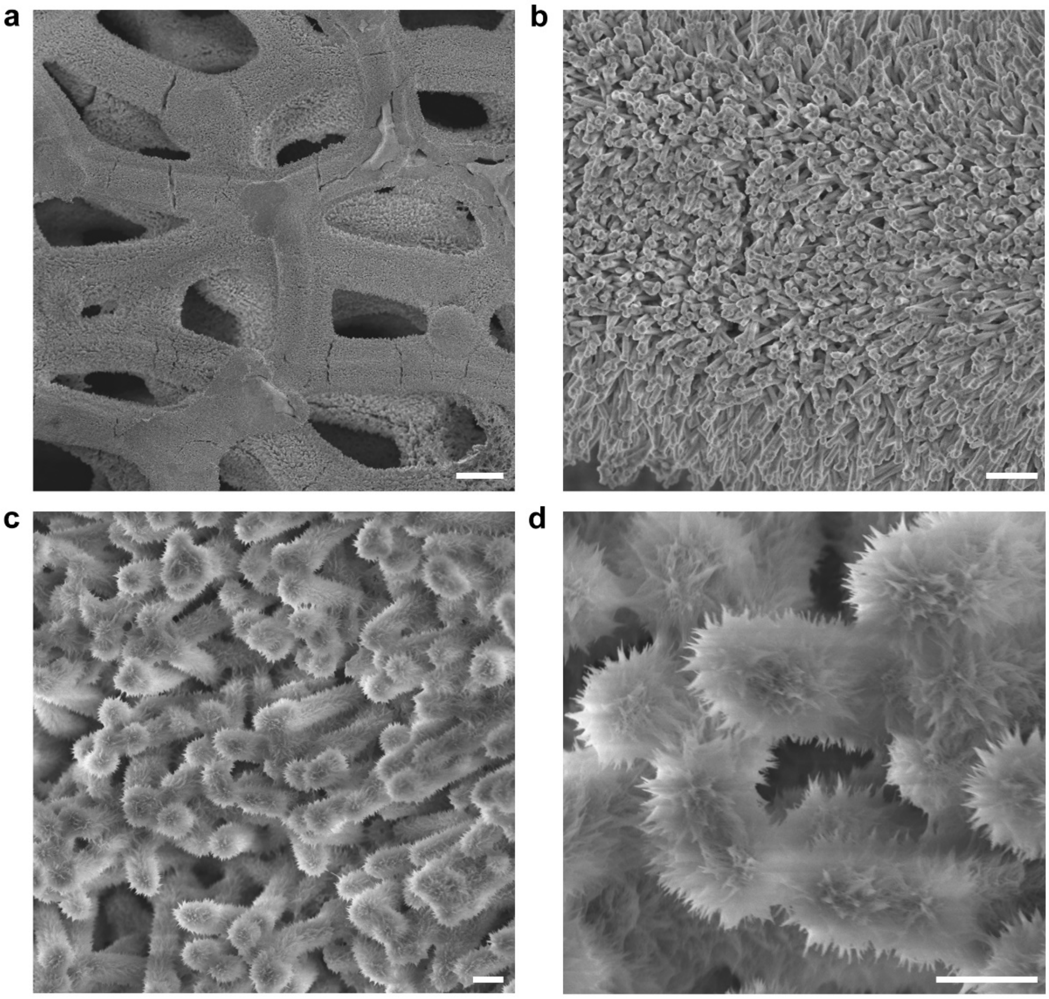

Supplementary Figure 3. SEM images of NiW-$Cu(OH)_2$ precursors. Scale bars, 100 μm (**a**), 10 μm (**b**), 1 μm (**c**-**d**).

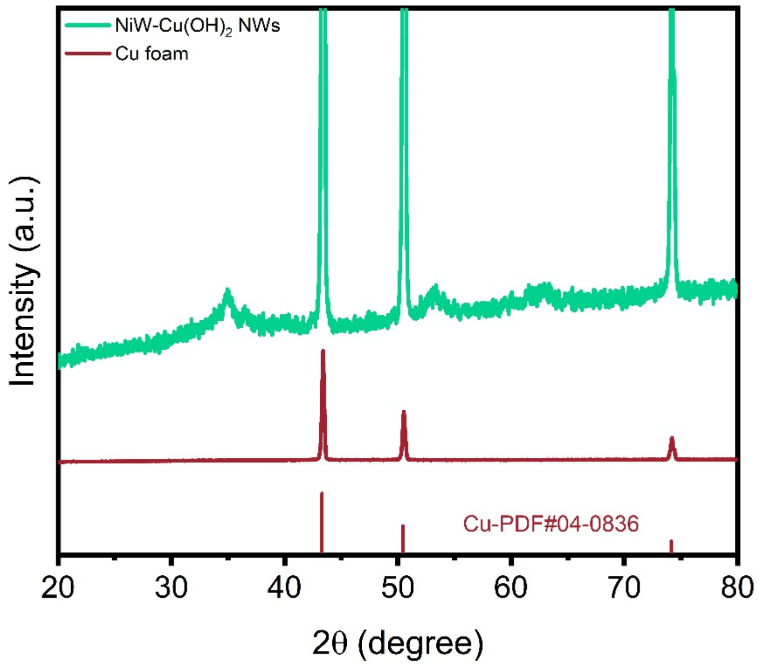

Supplementary Figure 4. XRD patterns of NiW-Cu(OH)$_2$ precursors and pure Cu foam.

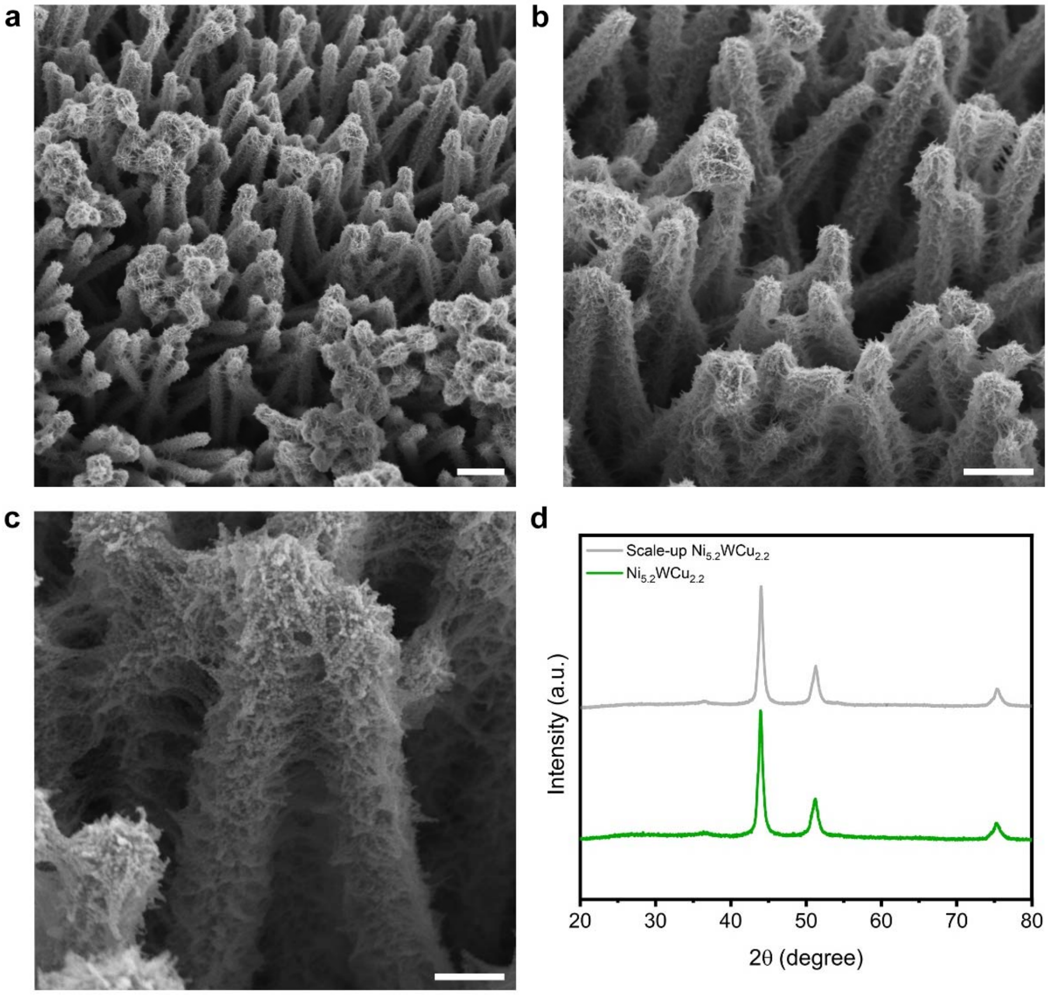

Supplementary Figure 5. Scaled-up synthesis of $Ni_{5.2}WCu_{2.2}$ alloy. a-c, SEM images. **d,** XRD spectra of the scaled-up $Ni_{5.2}WCu_{2.2}$ alloy. Scale bars, 1 μm (**a and b**), 200 nm (**c**).

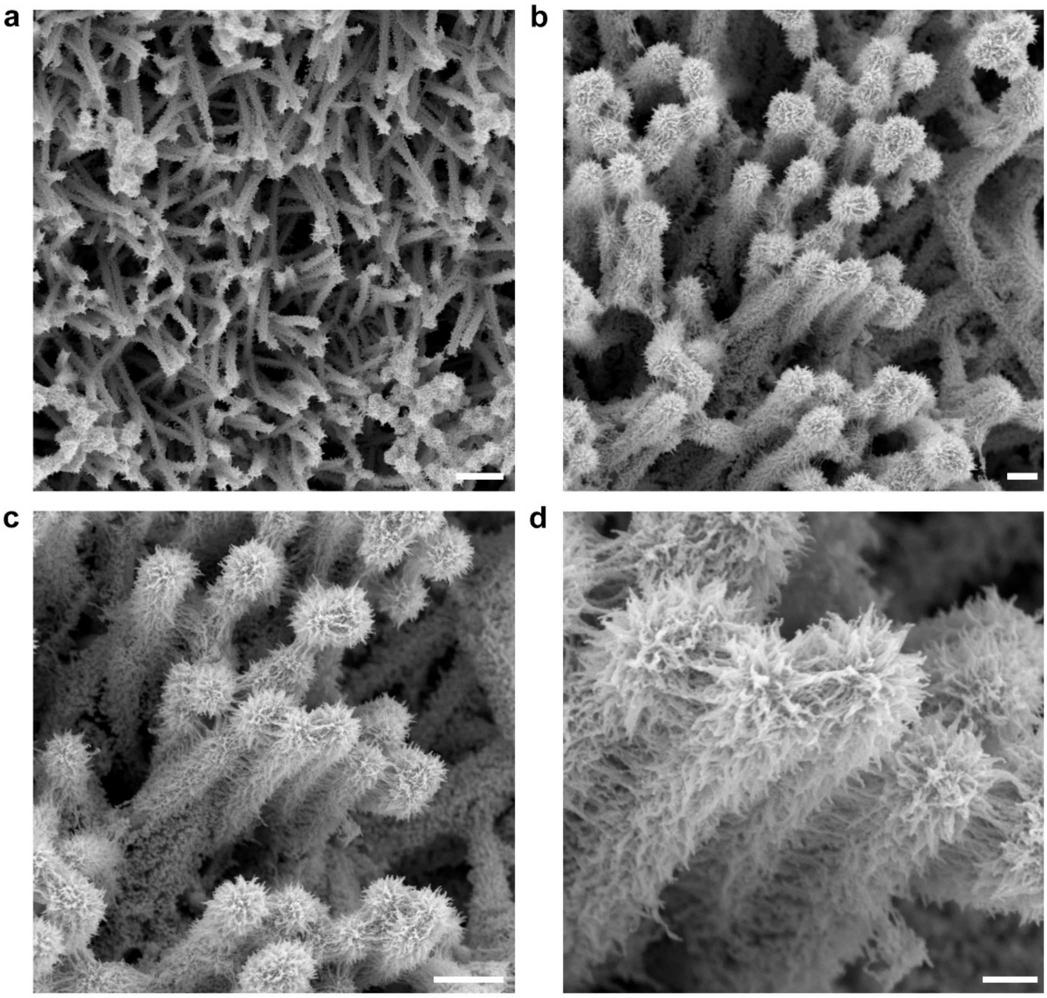

Supplementary Figure 6. Additional SEM images of ternary $Ni_{5.2}WCu_{2.2}$ alloy. Scale bars, 2 μm (**a**), 1 μm (**b**-**c**), 200 nm (**d**).

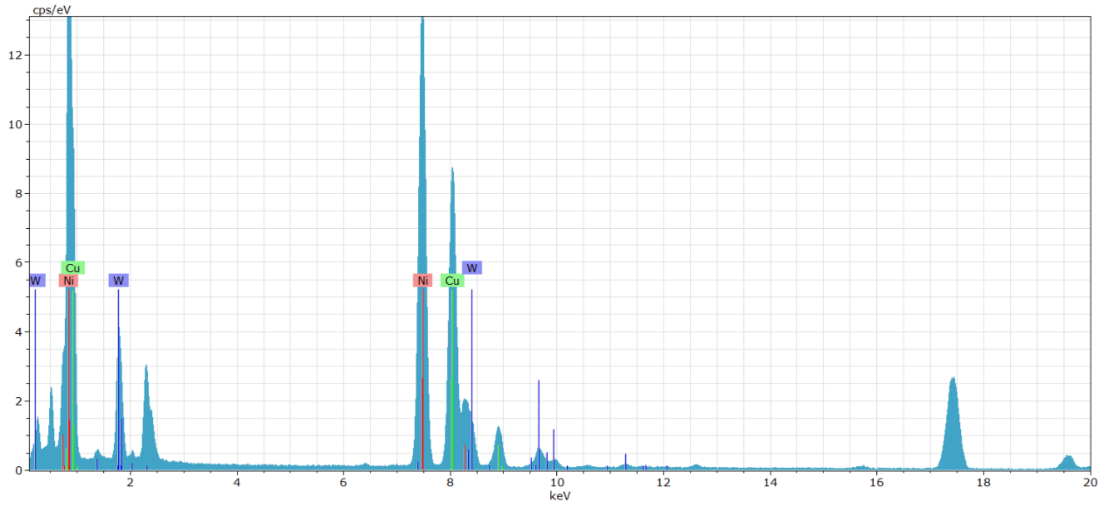

Supplementary Figure 7. EDX spectrum of the $Ni_{5.2}WCu_{2.2}$ alloy.

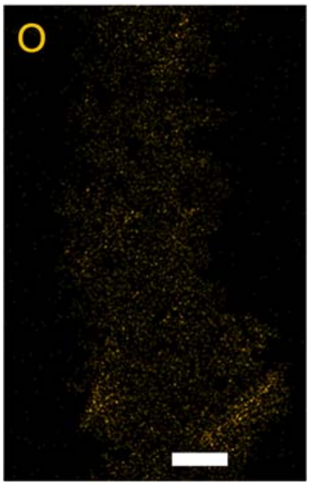

Supplementary Figure 8. EDX elemental mapping. It shows that O signal is very weak, probably originating from the adsorbed O and slight surface oxidation when exposing the sample in the air. Scale bar: 20 nm.

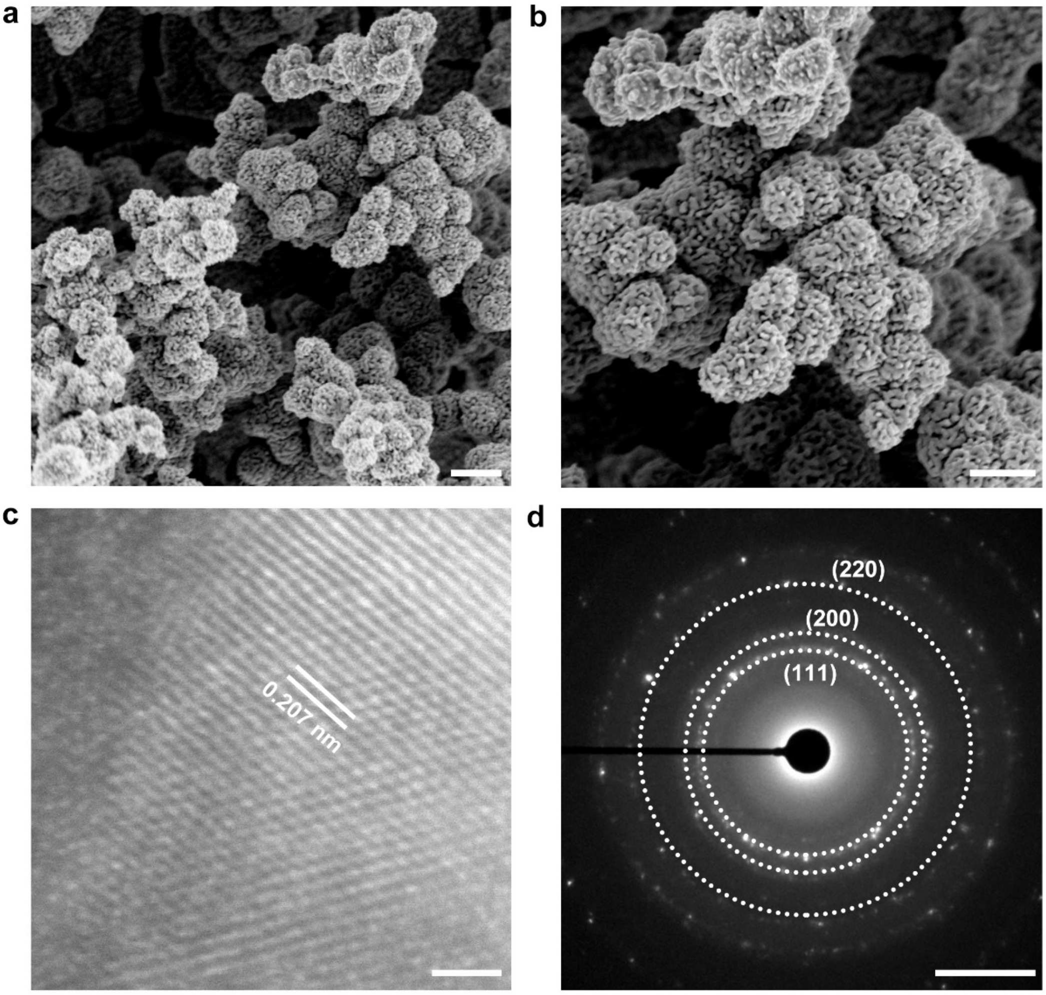

Supplementary Figure 9. Characterizations of $Ni_{17}W_3$. a-b, SEM images of as-synthesized $Ni_{17}W_3$. **c**, Atomic-resolution HAADF-STEM image of $Ni_{17}W_3$. **d**, Corresponding SAED pattern. Scale bars, 1 μm (**a**), 200 nm (**b**), 2 nm (**c**) and 5 1/nm (**d**).

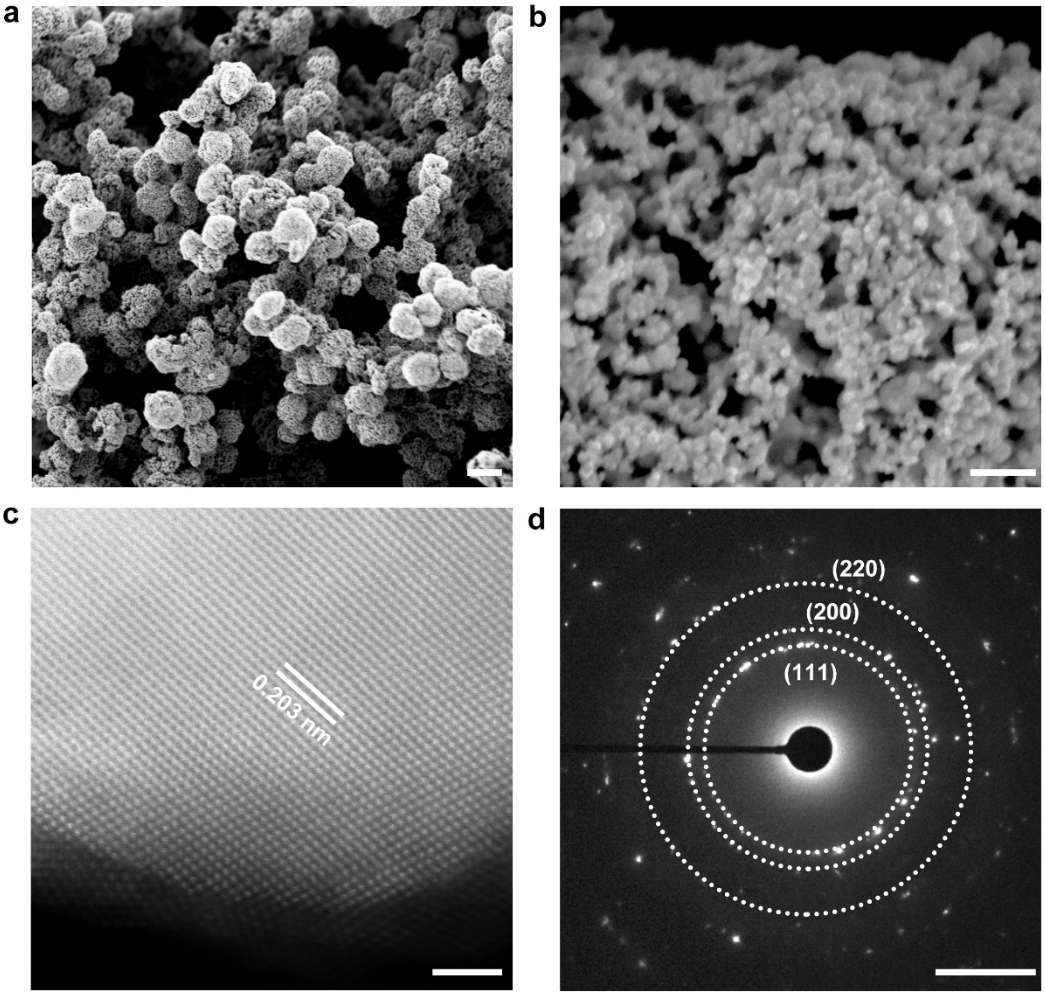

Supplementary Figure 10. Characterizations of as-synthesized Ni. a-b, SEM images of freshly-prepared Ni. **c**, Atomic-resolution HAADF-STEM image of Ni. **d**, SAED pattern. Scale bars, 2 μm (**a**), 200 nm (**b**), 2 nm (**c**), 5 1/nm (**d**).

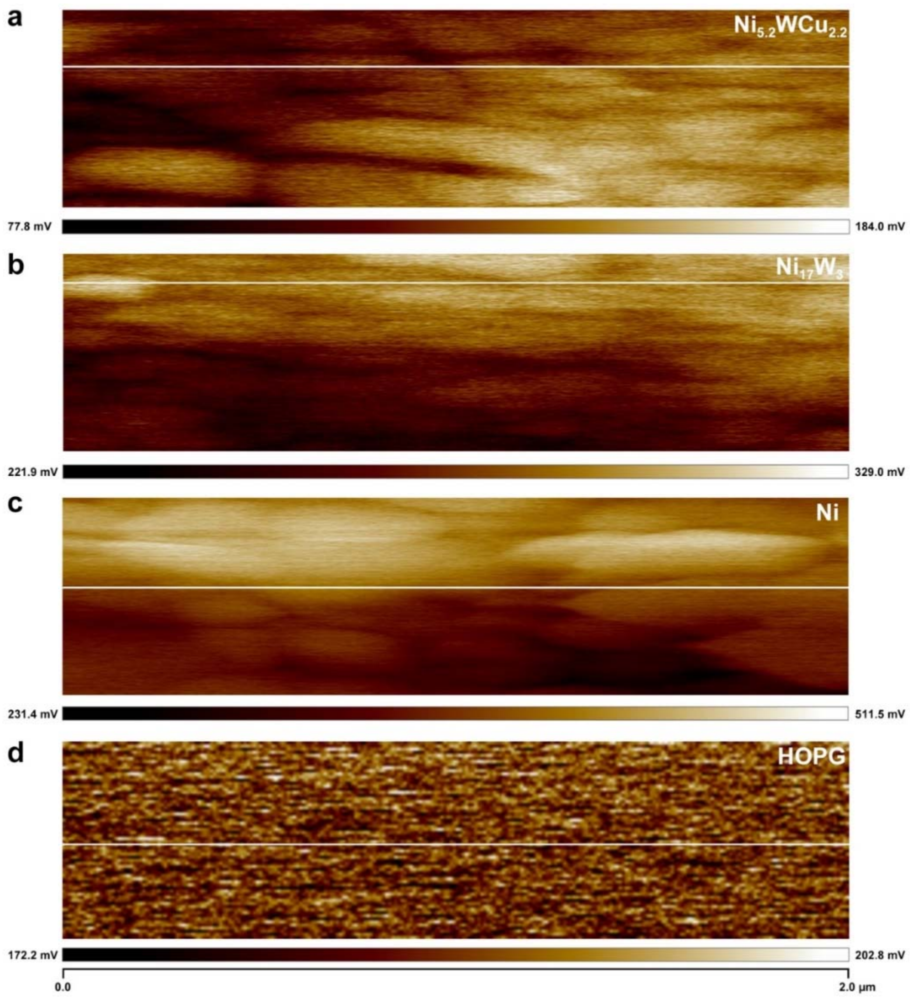

Supplementary Figure 11. The contact potential difference (CPD) images of $Ni_{5.2}WCu_{2.2}$, $Ni_{17}W_3$, Ni, and HOPG, respectively. The surface potentials in Figure 2e are derived from these CPD images along the white lines in each figures, which are 134 mV, 297 mV, 391 mV, and 190 mV for $Ni_{5.2}WCu_{2.2}$, $Ni_{17}W_3$, Ni, and HOPG, respectively. According to the potential differences between the catalysts and HOPG, their work functions can be calculated. Detailed information and principles about this method can be referred to Supplementary ref. 1.

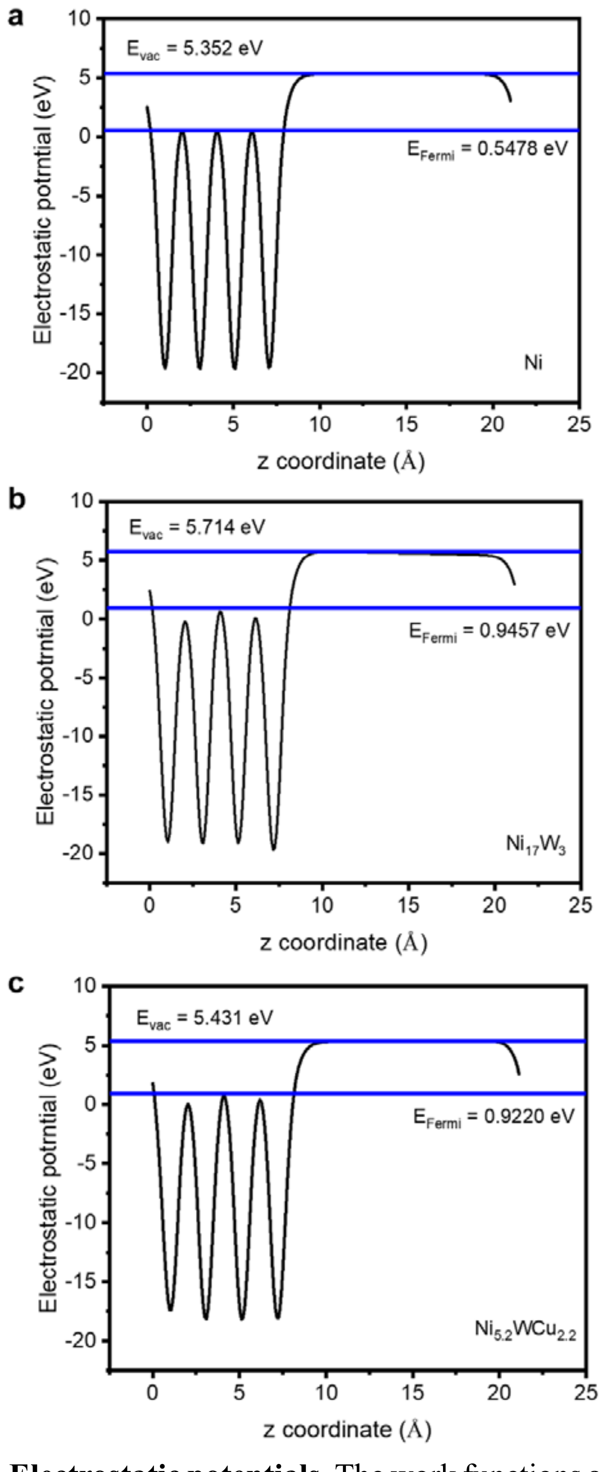

Supplementary Figure 12. Electrostatic potentials. The work functions are obtained via the equation: $\phi = E_{vac} - E_F$, where E_{vac} is the electrostatic potential of the vacuum level, and E_F is the Fermi energy. The work functions of the Ni(111), $Ni_{17}W_3$(111) and $Ni_{5.2}WCu_{2.2}$(111) surfaces were calculated to be 4.8042 eV, 4.7683eV, and 4.509 eV, respectively.

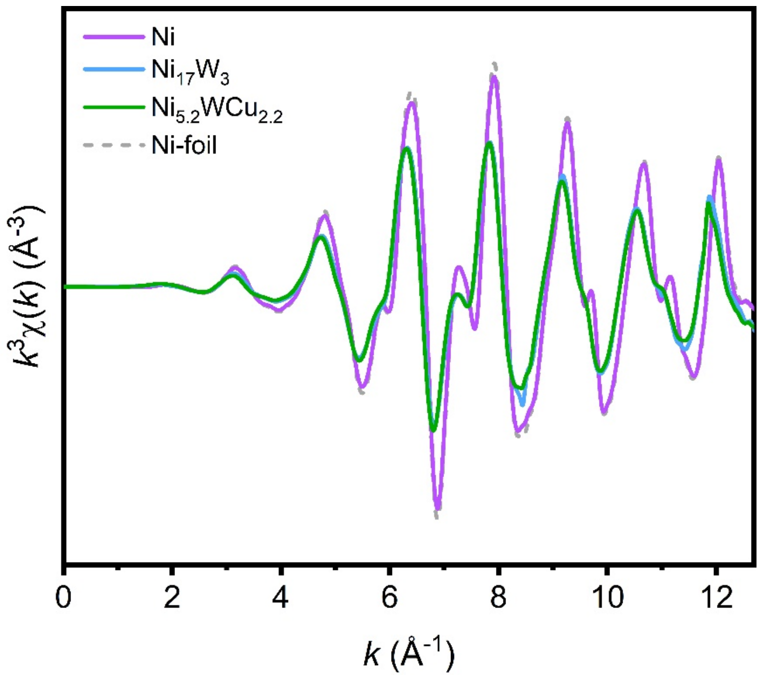

Supplementary Figure 13. Ni K-edge EXAFS oscillation spectra of freshly-synthesized Ni, $Ni_{17}W_3$, $Ni_{5.2}WCu_{2.2}$, and Ni foil reference.

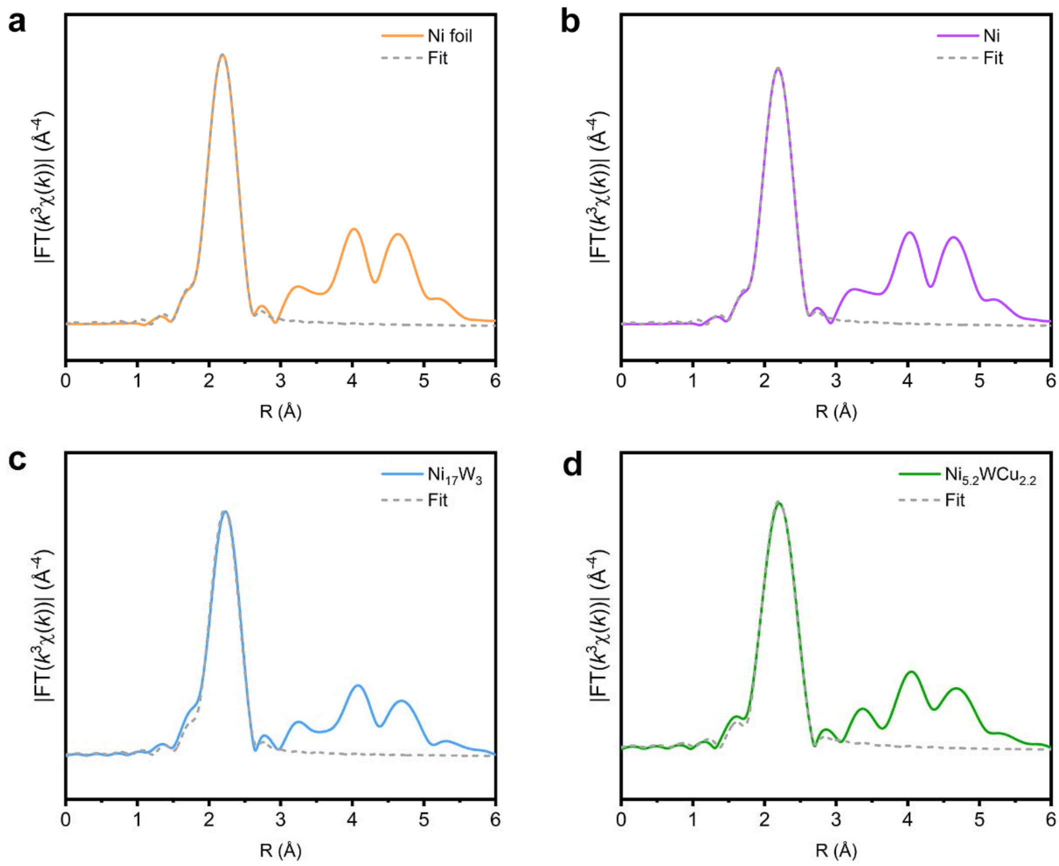

Supplementary Figure 14. Raw and fitting Fourier transform of k^3-weighted Ni K-edge EXAFS spectra. a, Ni foil. **b**, Ni. **c**, $Ni_{17}W_3$. **d**, $Ni_{5.2}WCu_{2.2}$. The first peak at about 2.1 Å in the EXAFS spectra can be attributed to the Ni-Ni or Ni-W(Cu) bonds[2-4]. The fitting results (Supplementary Table 1) show that the coordination numbers in the first coordination shell of Ni atoms for Ni foil, Ni, $Ni_{17}W_3$, and $Ni_{5.2}WCu_{2.2}$ are 12.0, 10.8, 8.7, and 9.0, respectively.

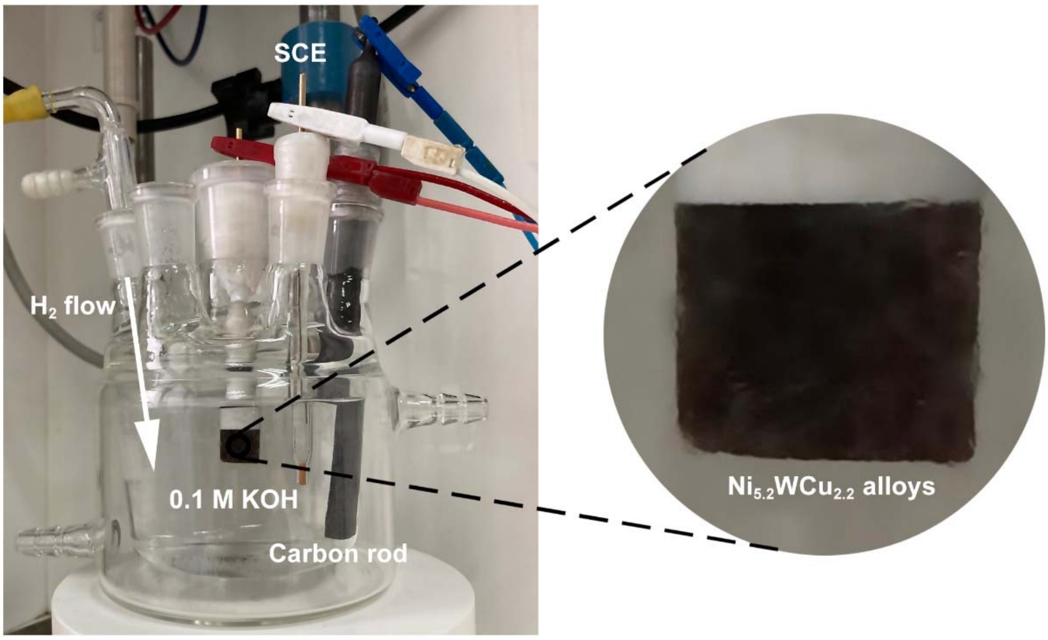

Supplementary Figure 15. HOR measurement setup. The $Ni_{5.2}WCu_{2.2}$ alloys were directly used as the working electrode in H_2-saturated 0.1 M KOH for electrochemical tests.

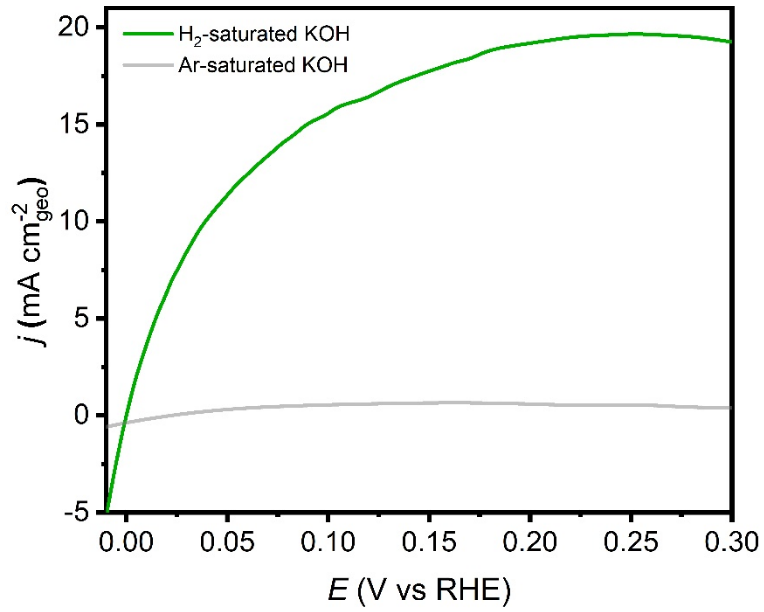

Supplementary Figure 16. HOR polarization curves of $Ni_{5.2}WCu_{2.2}$ in H_2- and Ar-saturated 0.1 M KOH. It can be seen that there is no HOR current in Ar-saturated KOH solution, indicating that anodic current in H_2-saturated KOH originates from the H_2 oxidation.

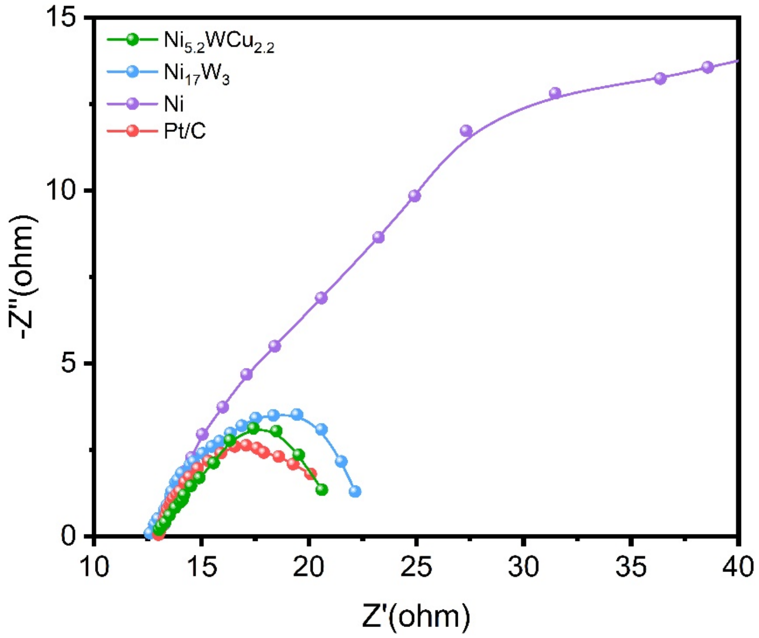

Supplementary Figure 17. EIS Nyquist plots of $Ni_{5.2}WCu_{2.2}$, $Ni_{17}W_3$, Ni, and Pt/C. The EIS are conducted in 0.1 M KOH under a 30 mV overpotential.

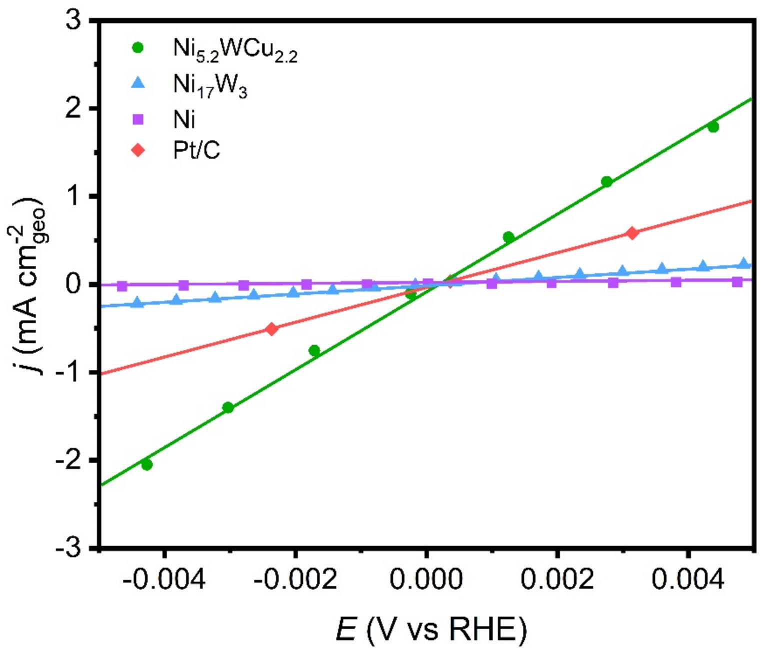

Supplementary Figure 18. Micro-polarization region (-5 mV to 5 mV) of $Ni_{5.2}WCu_{2.2}$, $Ni_{17}W_3$, Ni and Pt/C, respectively. The dashed lines indicate the linear fitting.

In the micro-polarization region, the Butler-Volmer equation can be simplified to

$$j_0 = \frac{j}{\eta}\frac{RT}{F} \quad (1)$$

where j is the measured current density, η is the overpotential, R is the universal gas constant, T is the temperature and F is the Faraday's constant. Thus, the exchange current density can be obtained from the slope of the linear fitting of j vs η. The obtained j_0 are consistent with the fitting results in Tafel regions (see Methods), which are listed in **Supplementary Table 2**.

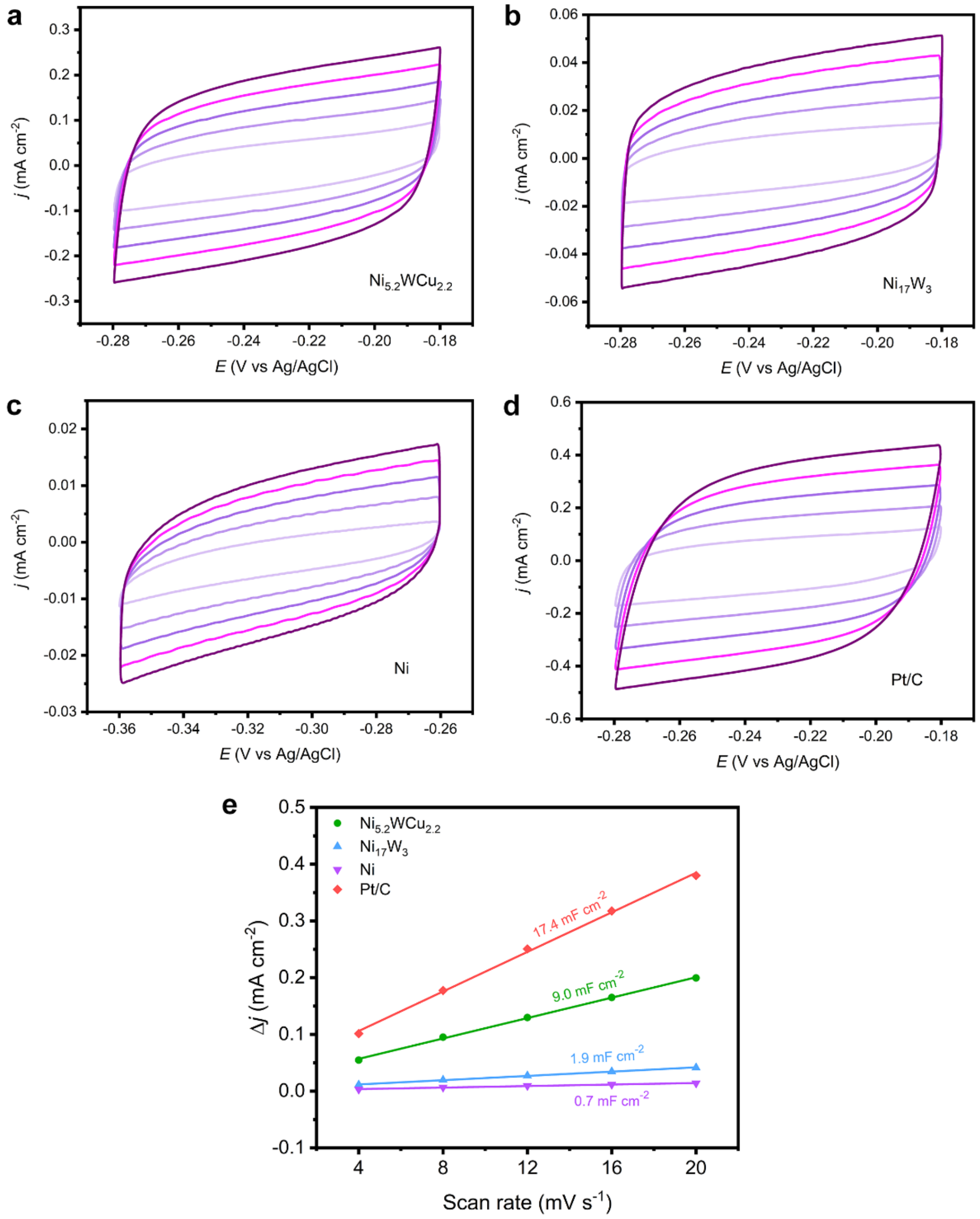

Supplementary Figure 19. ECSA measurements. a-d CV curves of $Ni_{5.2}WCu_{2.2}$ (**a**), $Ni_{17}W_3$ (**b**), Ni (**c**), and Pt/C (**d**) collected at various scan rates ranging from 4 to 20 mV s^{-1} in CH_3CN with 0.15 M KPF_6. **e** The linear fitting of scan rate versus Δj (the difference between the anodic and cathodic current densities at open circuit potential). We measure the ECSA of these catalysts by the double layer capacitance method on the basis of CV in a nonaqueous aprotic KPF_6–CH_3CN electrolyte[5]. The resulting linear slope is twice of the C_{dl} and the ECSA can be calculated according to the following equation: ECSA = C_{dl}/C_s, where C_s is the specific capacitance of a flat smooth surface of the electrode material, which was determined to be 11 μF cm^{-2}. The result gives an ECSA values of 818 cm^2 for $Ni_{5.2}WCu_{2.2}$, 173 cm^2 for $Ni_{17}W_3$, 64 cm^2 for Ni, and 1582 cm^2 for Pt/C, respectively.

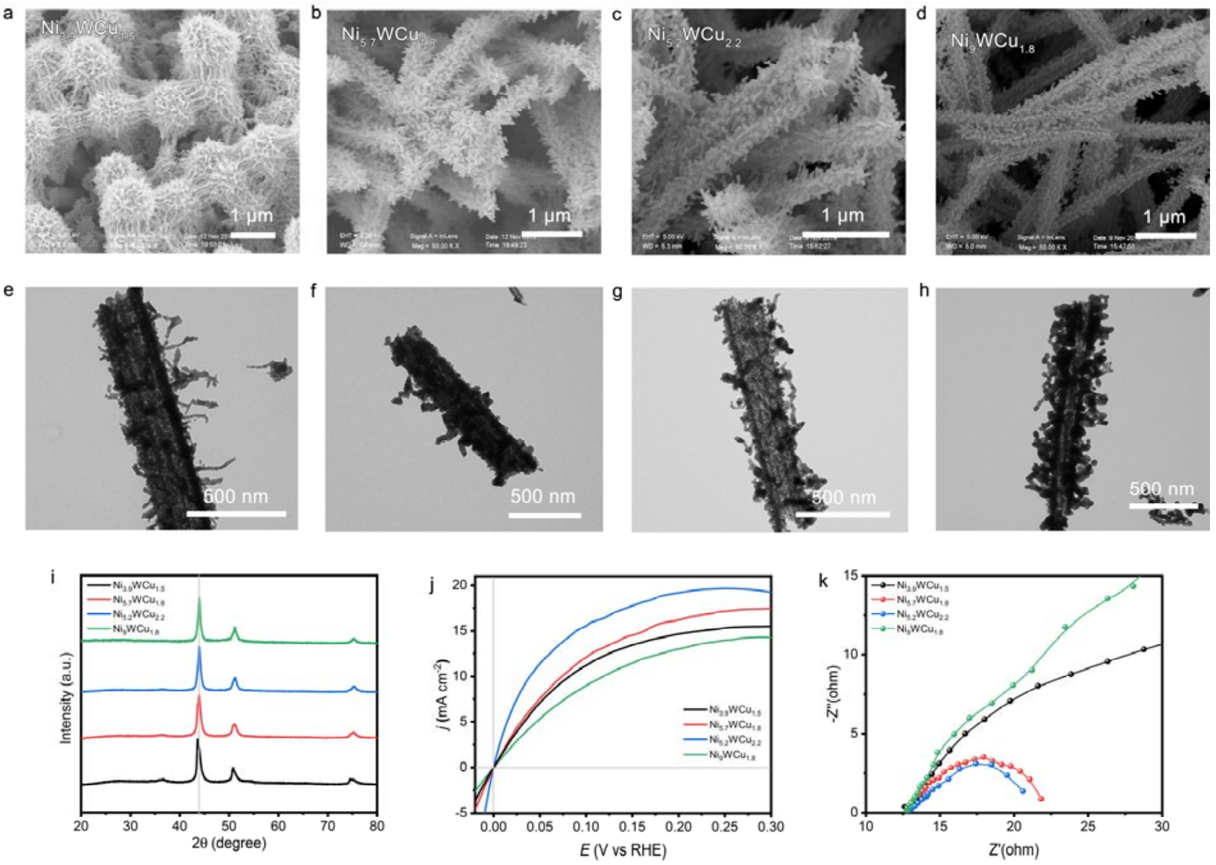

Supplementary Figure 20. Physical characterization and HOR activities of Ni-W-Cu alloys with different ratios. a-h, SEM and TEM images of catalysts obtained under different addition amounts of Ni and W precursors: $Ni_{3.9}WCu_{1.5}$ (**a, e**), $Ni_{5.7}WCu_{1.8}$ (**b, f**), $Ni_{5.2}WCu_{2.2}$ (**c, g**), $Ni_9WCu_{1.8}$ (**d, h**). **i**, XRD patterns. **j**, HOR polarization curves. **k**, EIS Nyquist plots. The catalyst with Ni/W/Cu ratio of 5.2:1:2.2 shows the best HOR performance. All the samples are obtained by annealing at 500 °C for 1.0 h.

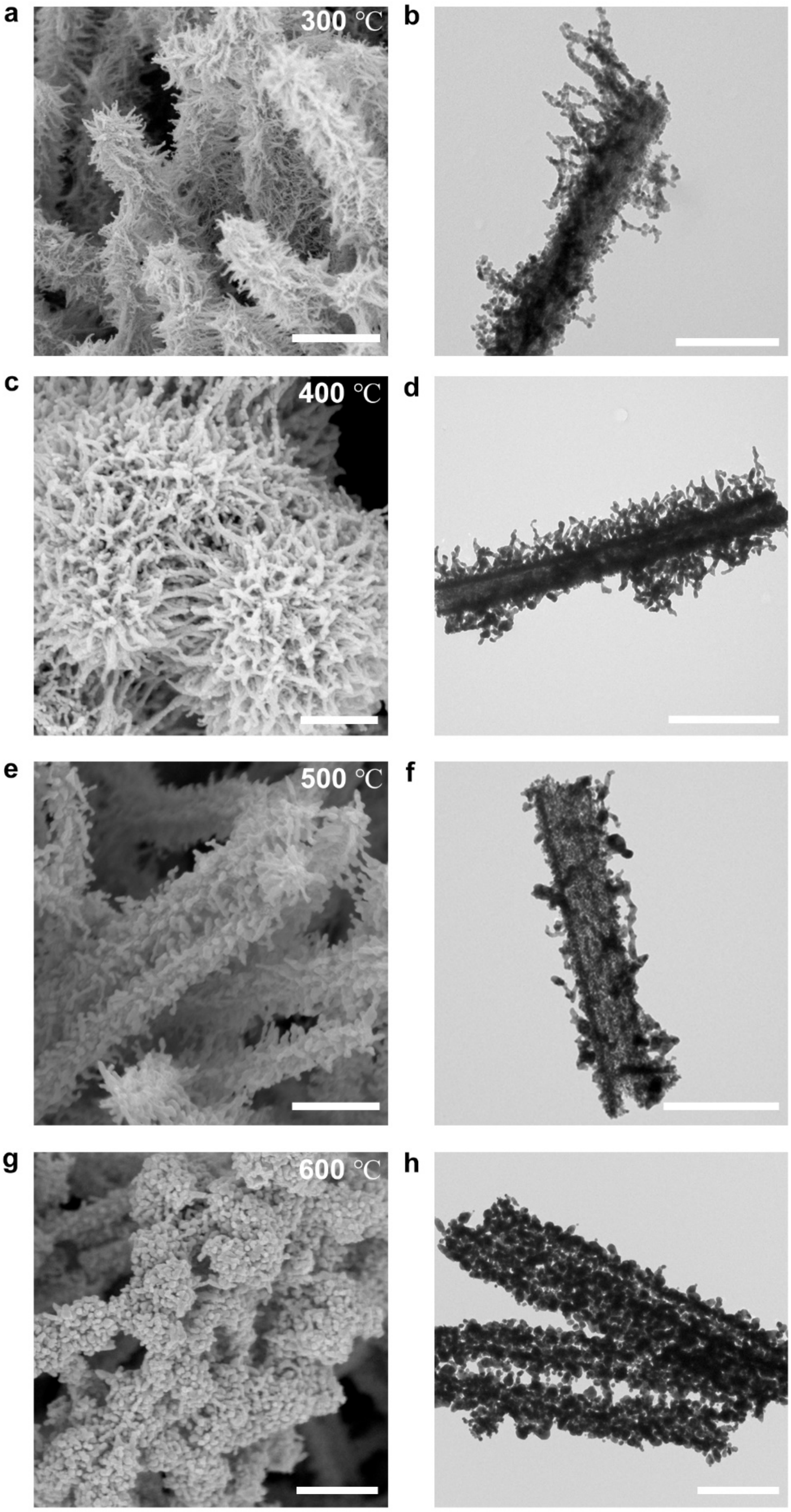

Supplementary Figure 21. Characterization of $Ni_{5.2}WCu_{2.2}$ alloys obtained at different temperatures for 1 h. a-h, SEM and TEM images of catalysts obtained at 300 ℃ (**a, b**), 400 ℃ (**c, d**), 500 ℃ (**e, f**), 600 ℃ (**g, h**). Scale bars, 500 nm.

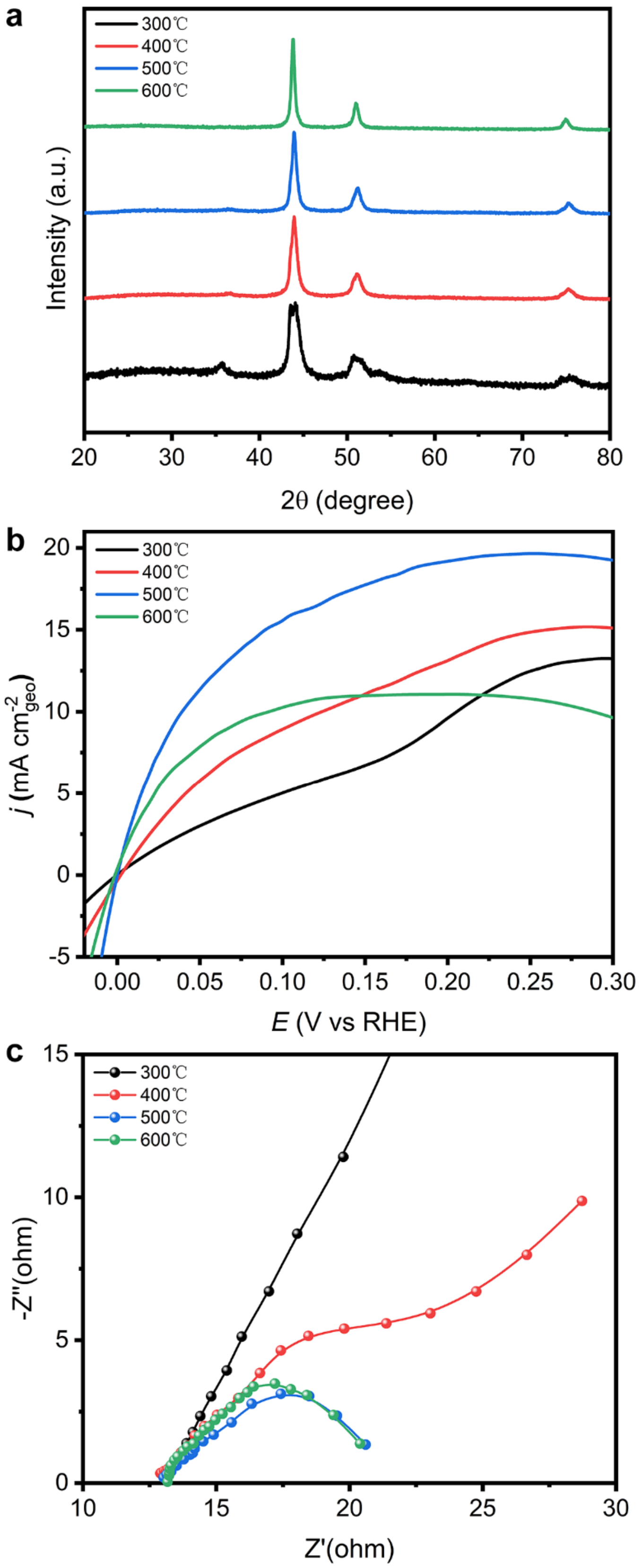

Supplementary Figure 22. Characterization and HOR activities of $Ni_{5.2}WCu_{2.2}$ alloys obtained at different temperatures for 1 h. a, XRD patterns. **b**, HOR polarization curves. **c**, EIS Nyquist plots. The catalyst reduced at 500 °C shows the best HOR performance (**b**) and the lowest charge transfer resistance (**c**).

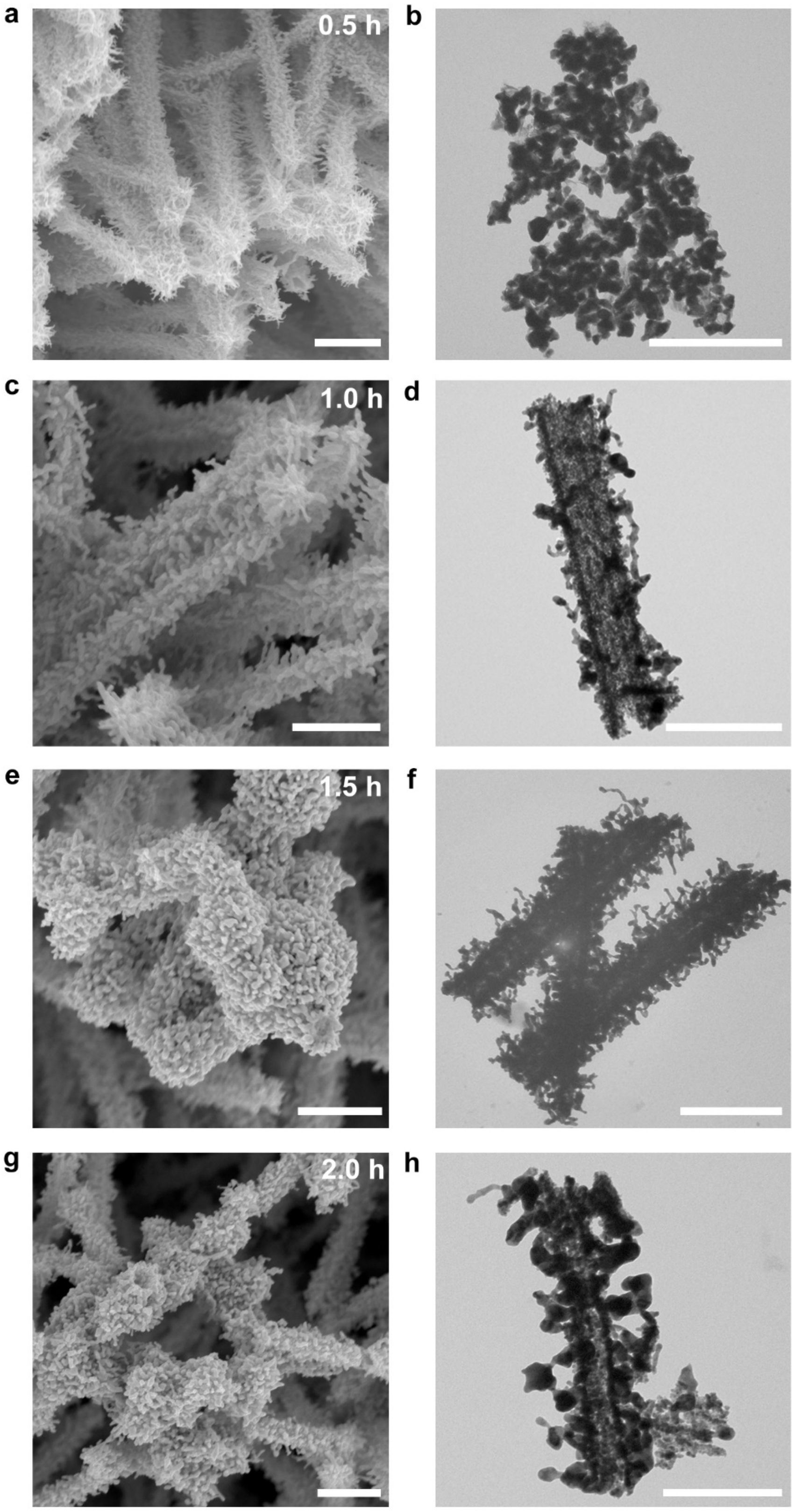

Supplementary Figure 23. Characterization of $Ni_{5.2}WCu_{2.2}$ alloys obtained at 500 °C for different times. a-h, SEM and TEM images of catalysts synthesized for 0.5 h (**a, b**), 1.0 h (**c, d**), 1.5 h (**e, f**), 2.0 h (**g, h**). Scale bars, 500 nm.

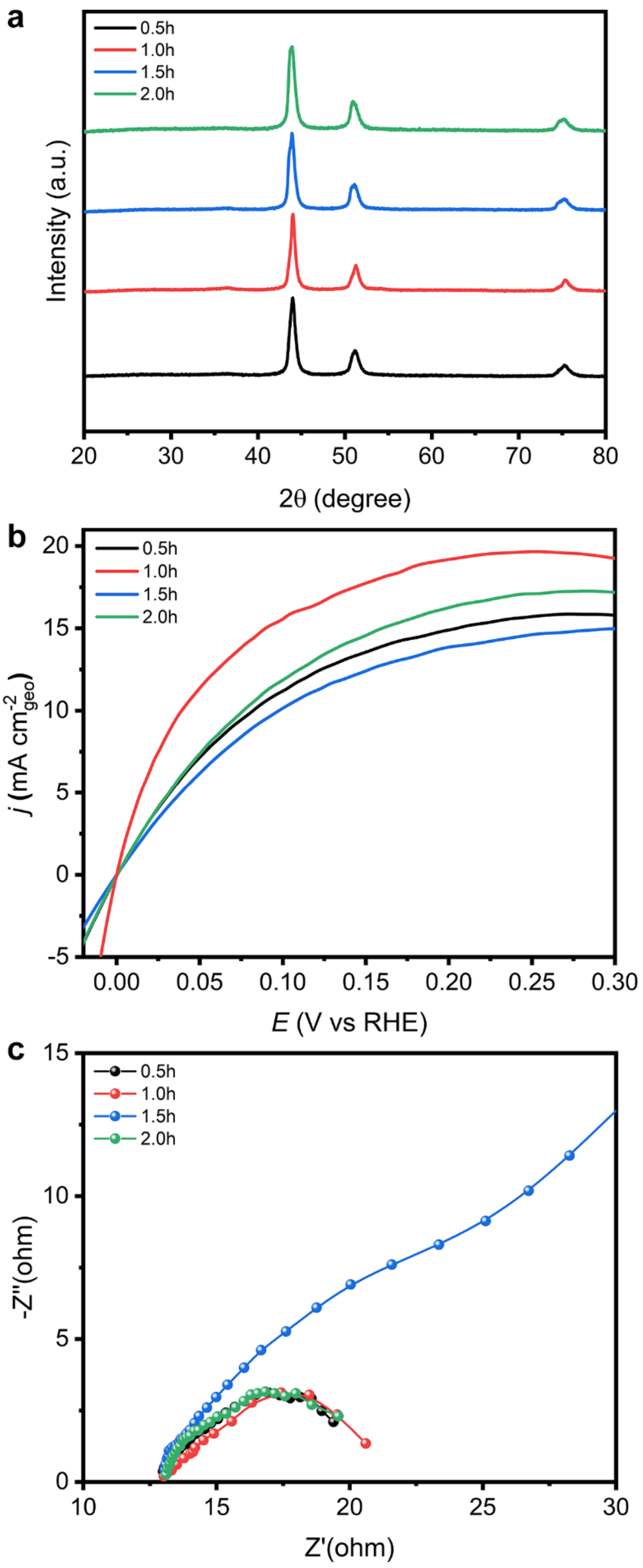

Supplementary Figure 24. Characterization and HOR activities of $Ni_{5.2}WCu_{2.2}$ alloys obtained at 500 °C for different times. a, XRD patterns. **b**, HOR polarization curves. **c**, EIS Nyquist plots. The catalyst reduced for 1 h shows the best HOR performance (**b**) and the lowest charge transfer resistance (**c**).

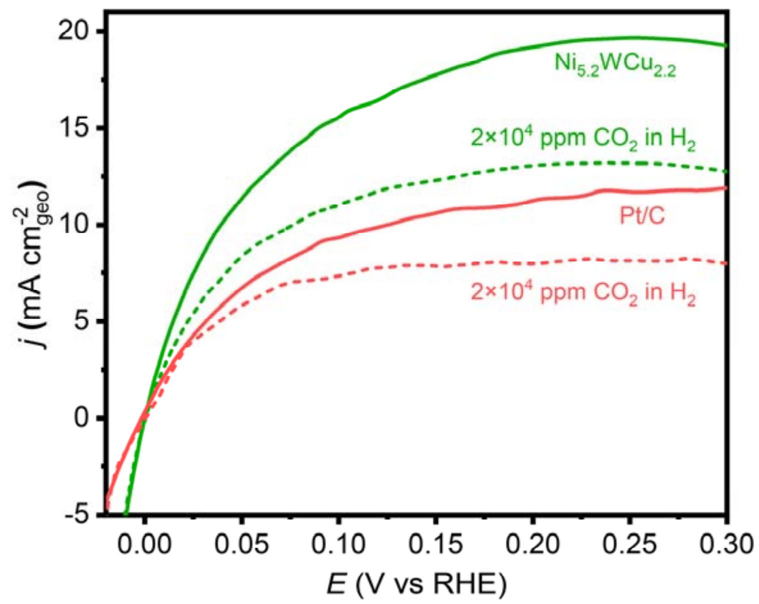

Supplementary Figure 25. HOR polarization curves for $Ni_{5.2}WCu_{2.2}$ alloy and Pt/C in H_2-saturated 0.1 M KOH with (dashed lines) and without (solid lines) the presence of 20,000 ppm CO_2.

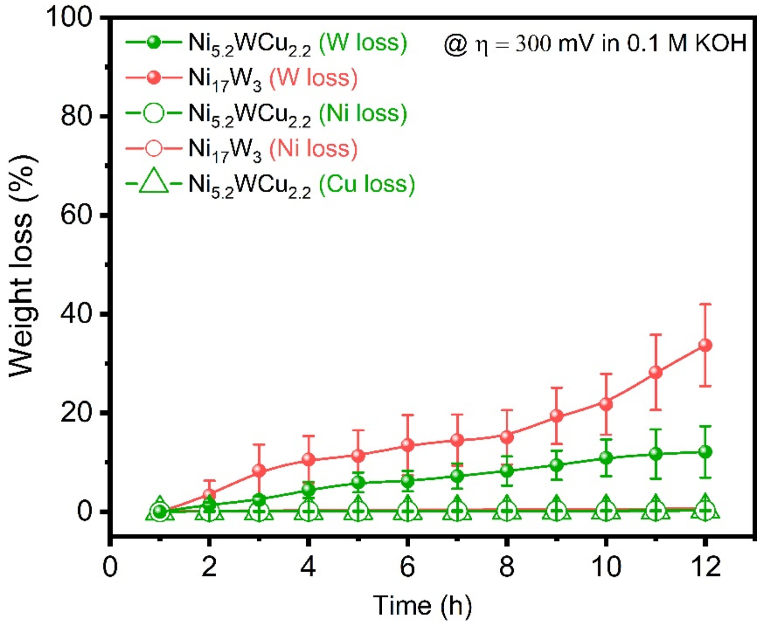

Supplementary Figure 26. Electrochemical leaching of Ni, W, Cu after different operating times. The ICP-AES results compare Ni, W, Cu leaching from $Ni_{17}W_3$ and $Ni_{5.2}WCu_{2.2}$ under 0.3 V overpotential in 0.1 M KOH. It shows lower W weight loss from $Ni_{5.2}WCu_{2.2}$ than that from $Ni_{17}W_3$ after 12 h durability test. Ni and Cu loss from both samples are negligible. These results indicate a better stability of $Ni_{5.2}WCu_{2.2}$ than $Ni_{17}W_3$.

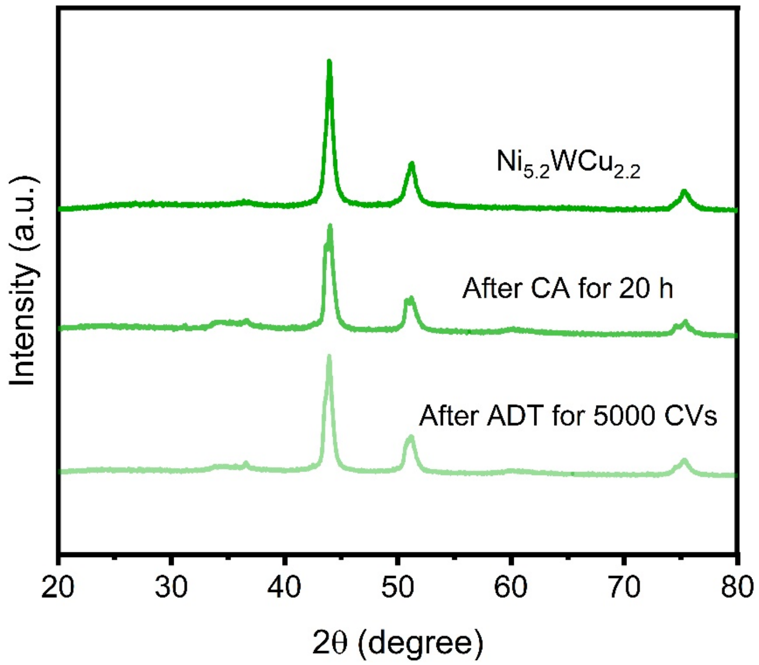

Supplementary Figure 27. XRD patterns of $Ni_{5.2}WCu_{2.2}$ after long-term stability test. It shows that the crystal phase of $Ni_{5.2}WCu_{2.2}$ remains unchanged after long-time stability measurements.

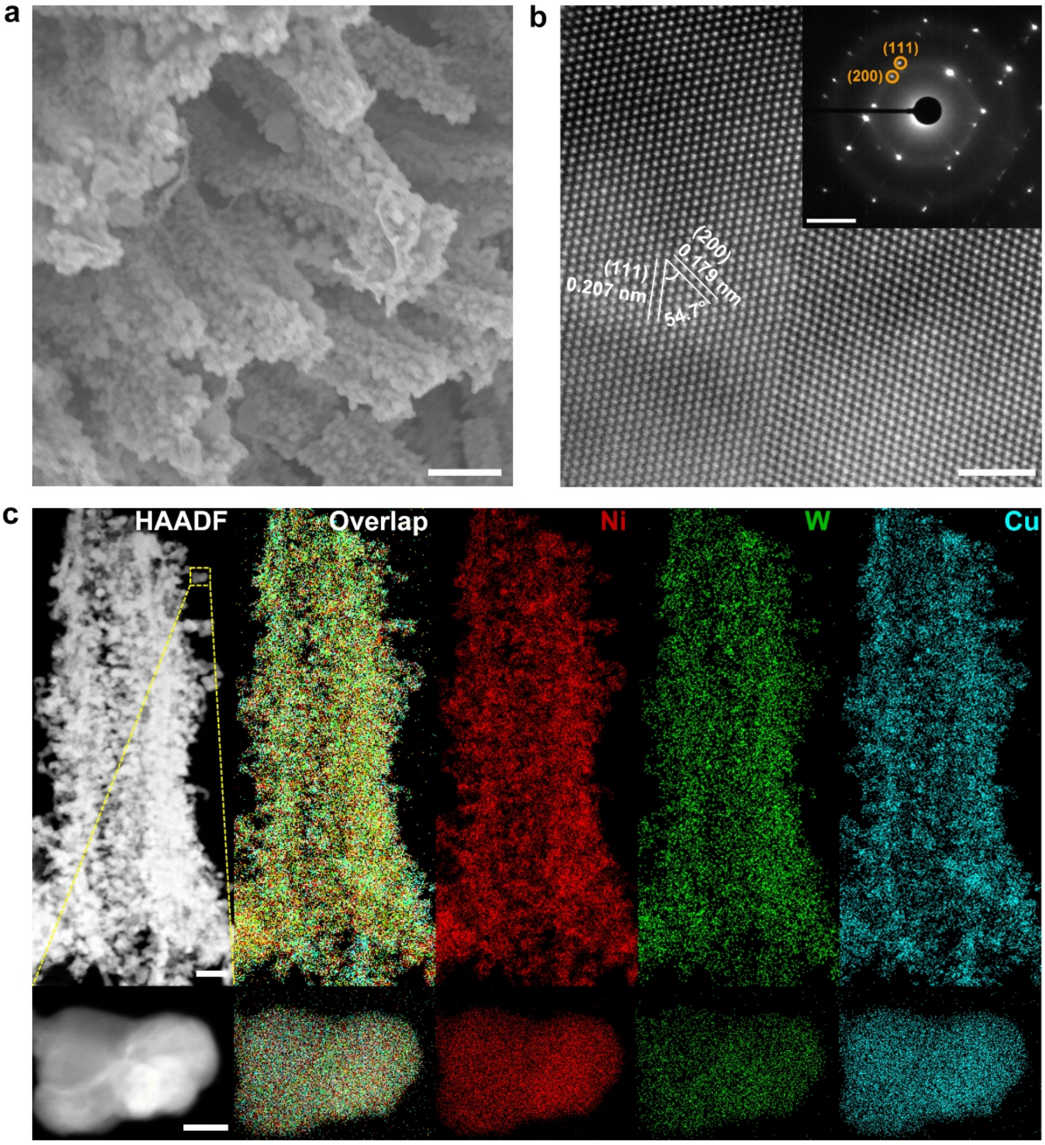

Supplementary Figure 28. Element and structural analysis of $Ni_{5.2}WCu_{2.2}$ after ADT in 0.1 M KOH. a, SEM image of $Ni_{5.2}WCu_{2.2}$ after ADT. **b**, Atomic-resolution HAADF-STEM image of $Ni_{5.2}WCu_{2.2}$ after ADT. Inset in **b** gives corresponding SAED pattern. **c**, STEM-EDX elemental mapping shows the morphology of $Ni_{5.2}WCu_{2.2}$ alloys and distribution of Ni, W, Cu elements are well retained after ADT. The ADT was carried out between -0.2 V to 0.2 V at a scan rate of 200 mV s^{-1} for 5,000 CV cycles. Scale bars, 1 μm (**a**), 2 nm (**b**), 5 1/nm (inset in **b**), 100 nm (up in **c**) and 10 nm (down in **c**).

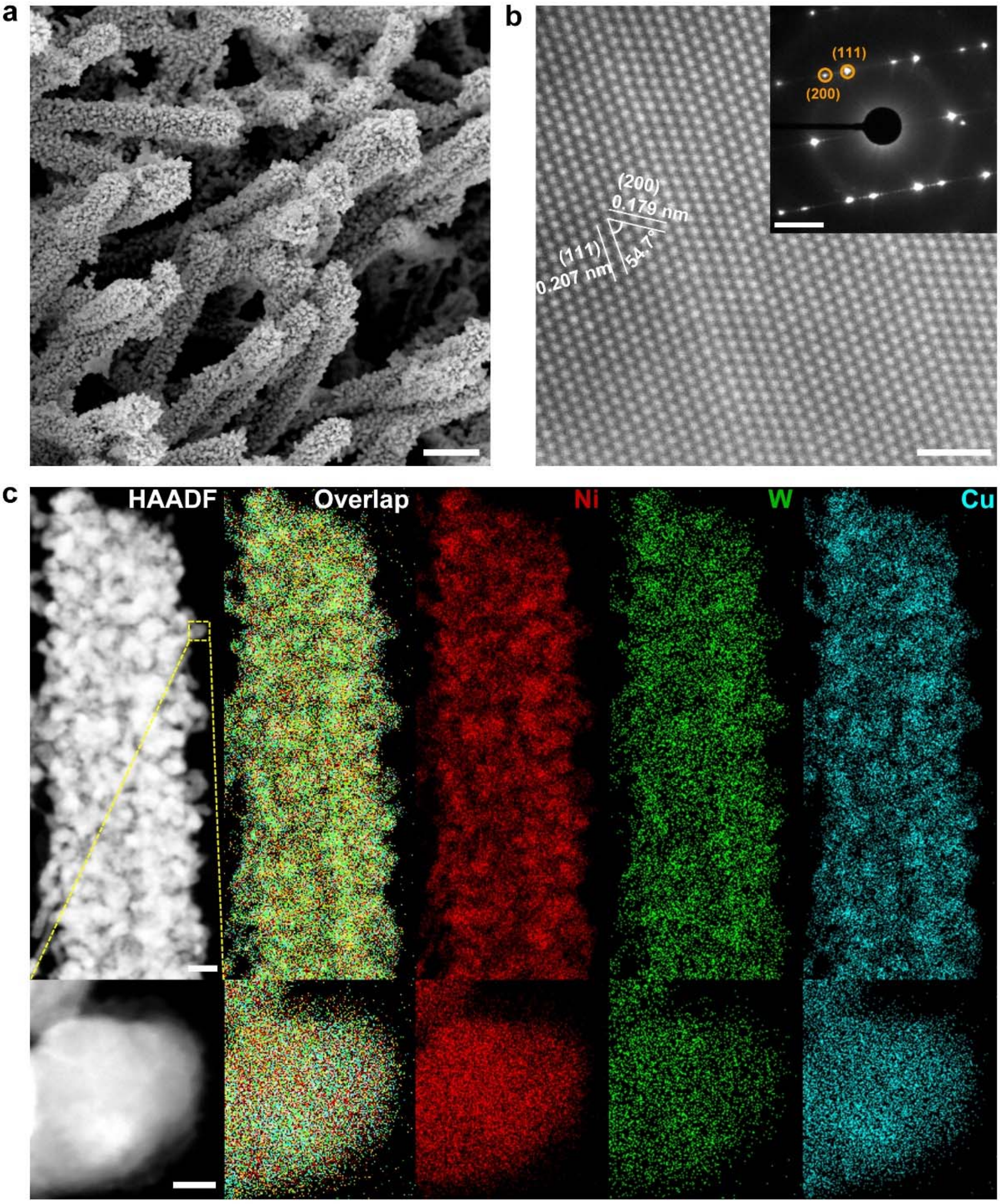

Supplementary Figure 29. Element and structural analysis of $Ni_{5.2}WCu_{2.2}$ after 20 h CA stability test at 0.3 V overpotential in 0.1 M KOH. a, SEM image of $Ni_{5.2}WCu_{2.2}$ after stability test. **b**, Atomic-resolution HAADF-STEM image of $Ni_{5.2}WCu_{2.2}$ after stability test. Inset in **b** gives corresponding SAED pattern. **c**, STEM-EDX elemental mapping shows the morphology of $Ni_{5.2}WCu_{2.2}$ alloys and distribution of Ni, W, Cu elements are well retained after long-time stability test. Scale bars, 1 μm (**a**), 2 nm (**b**), 5 1/nm (inset in **b**), 100 nm (up in **c**) and 10 nm (down in **c**).

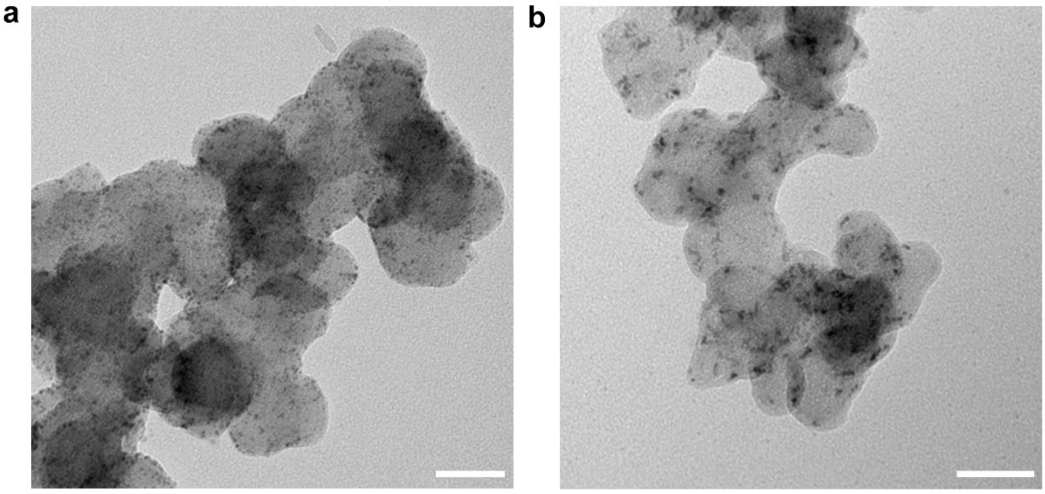

Supplementary Figure 30. TEM images of commercial Pt/C before and after 20 h CA stability test. a, TEM image of Pt/C before stability test. **b**, TEM image of Pt/C after stability test. The CA was carried out in 0.1 M H_2-saturated KOH at 0.3 V overpotential. Scale bars, 50 nm. As shown, the Pt particle obviously grows and forms agglomerated clusters after long-time stability test, which results in reduction of active surface areas and further activity degradation, in agreement with previous reports on Pt/C catalyst[6,7].

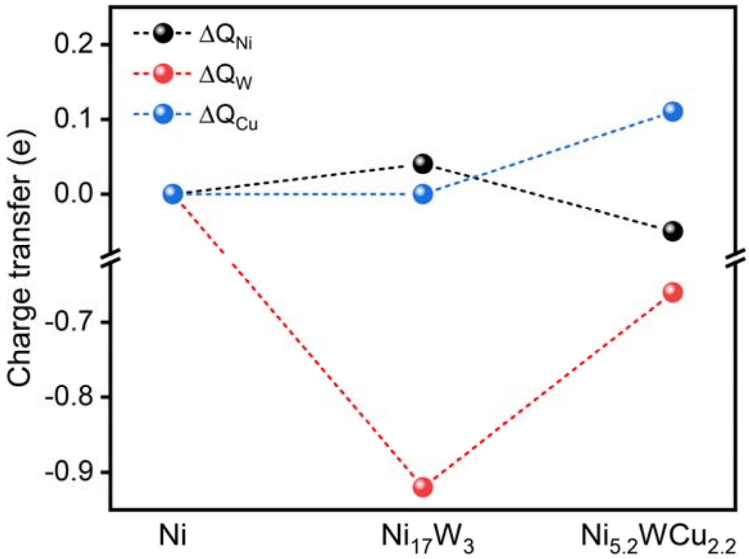

Supplementary Figure 31. The Bader charge analysis. The positive values represent the atoms get electrons from other atoms, whereas the negative values indicate electron donation to other atoms.

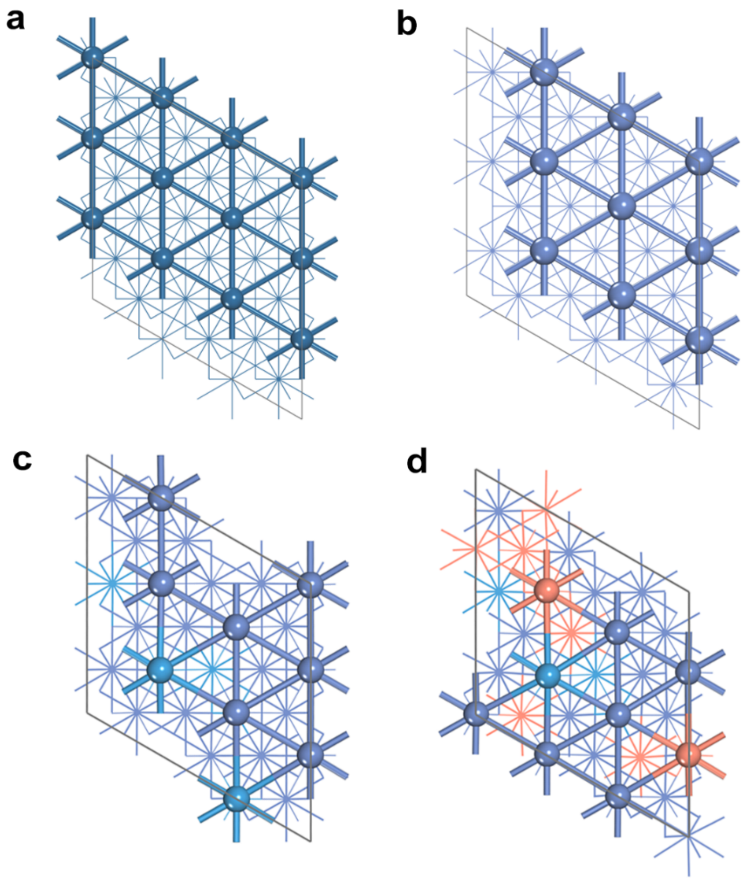

Supplementary Figure 32. Constructed models. **a**, Pt(111). **b**, Ni(111). **c**, $Ni_{17}W_3$(111). **d**, $Ni_{5.2}WCu_{2.2}$(111). The darkblue, violet, blue, and orange spheres represent Pt, Ni, W, and Cu atoms, respectively.

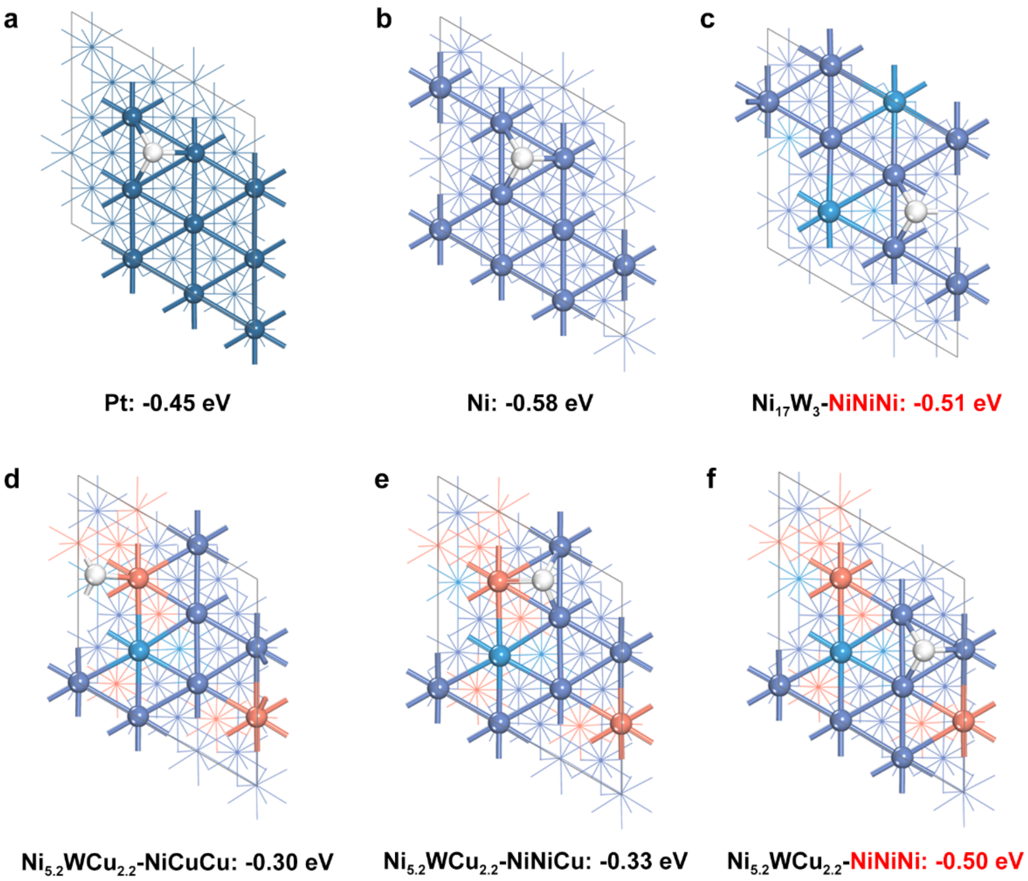

Supplementary Figure 33. Models of H adsorption on catalysts. **a**, Pt(111). **b**, Ni(111). **c**, $Ni_{17}W_3$(111). **d**, $Ni_{5.2}WCu_{2.2}$(111). The darkblue, violet, blue, orange, and white spheres represent Pt, Ni, W, Cu, and H atoms, respectively. The red-marked adsorption sites stand for the most stable models.

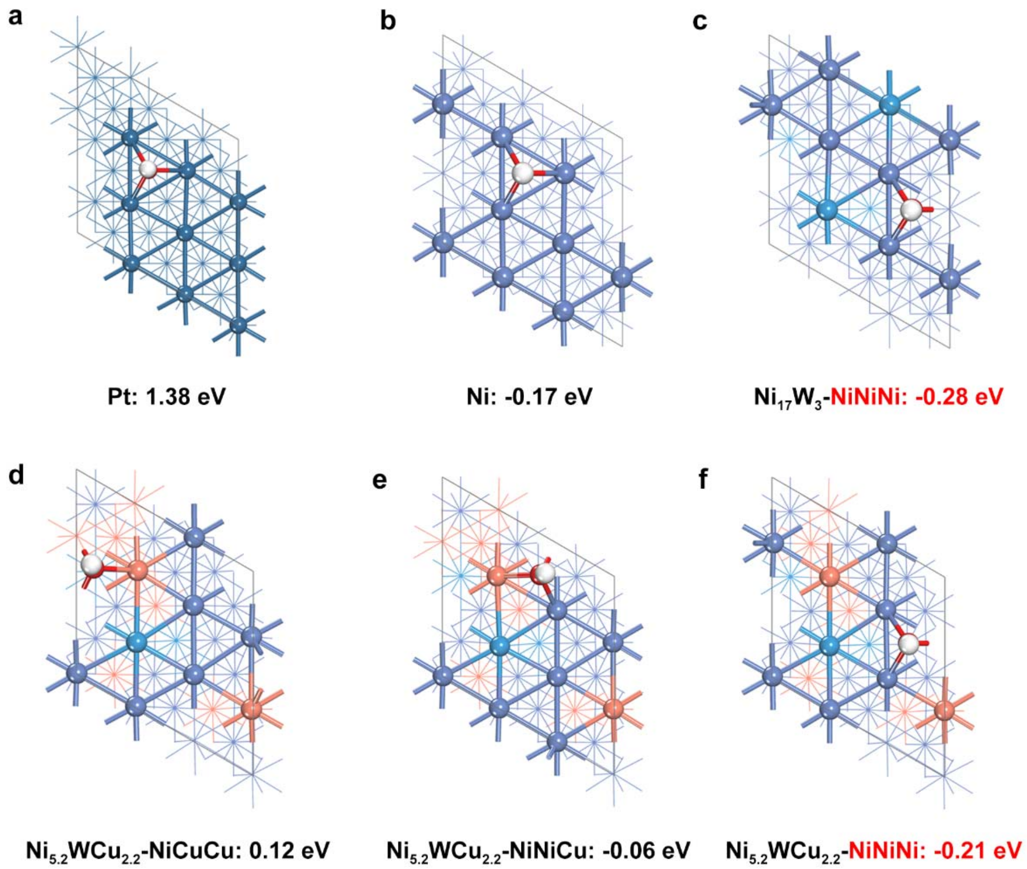

Supplementary Figure 34. Models of OH adsorption on catalysts. a, Pt(111). **b**, Ni(111). **c**, $Ni_{17}W_3$(111). **d**, $Ni_{5.2}WCu_{2.2}$(111). The darkblue, violet, blue, orange, white, and red spheres represent Pt, Ni, W, Cu, H, and O atoms, respectively. The red-marked adsorption sites stand for the most stable models.

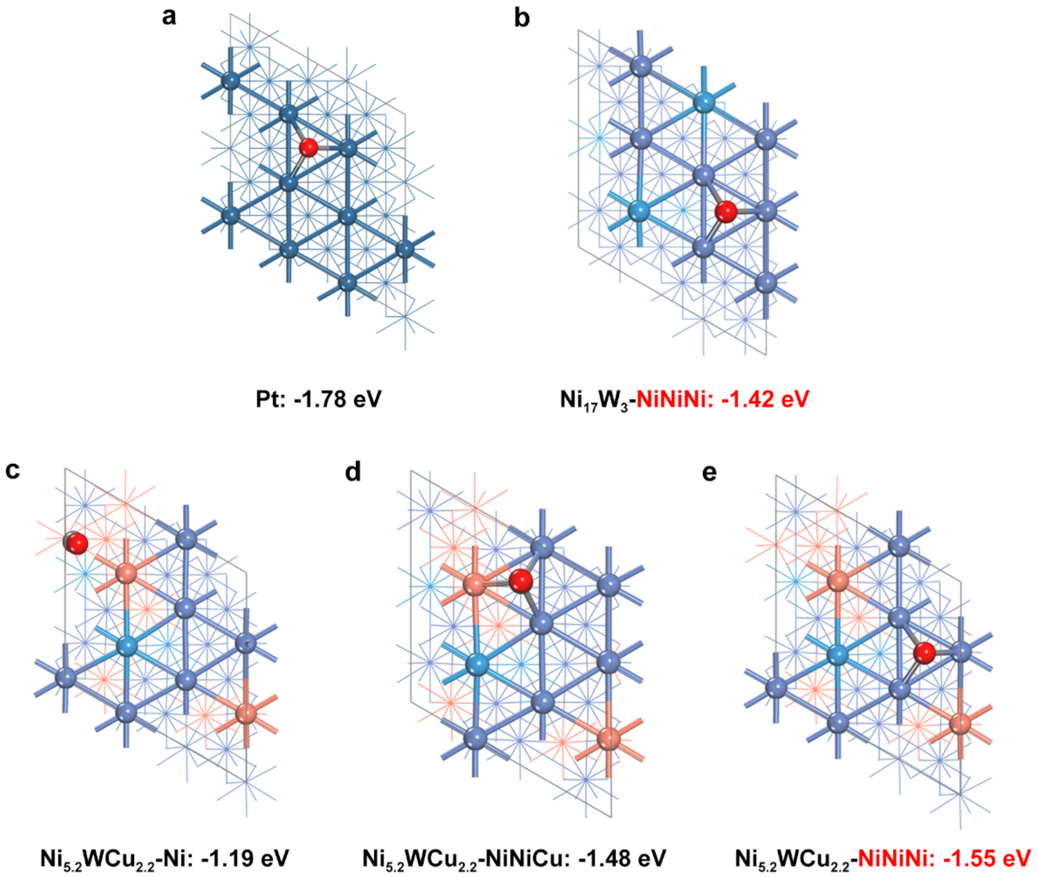

Supplementary Figure 35. Models of CO adsorption on catalysts. a, Pt(111). **b**, $Ni_{17}W_3$(111). **c**, $Ni_{5.2}WCu_{2.2}$(111). The darkblue, violet, blue, orange, black, and red spheres represent Pt, Ni, W, Cu, C, and O atoms, respectively. The red-marked adsorption sites stand for the most stable models.

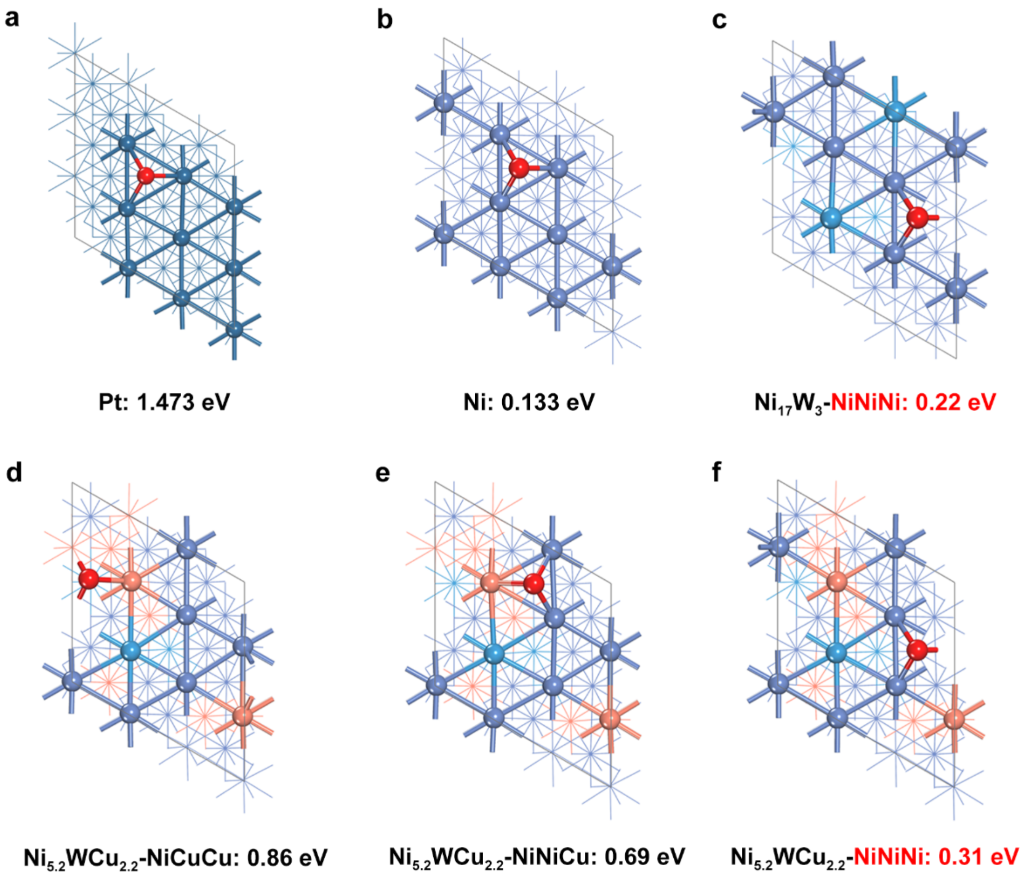

Supplementary Figure 36. Models of O adsorption on catalysts. a, Pt(111). **b**, Ni(111). **c**, $Ni_{17}W_3$(111)-NiNiNi. **d**, $Ni_{5.2}WCu_{2.2}$(111)-NiCuCu. e, $Ni_{5.2}WCu_{2.2}$(111)-NiNiCu. f, $Ni_{5.2}WCu_{2.2}$(111)-NiNiNi. The darkblue, violet, blue, orange, and red spheres represent Pt, Ni, W, Cu, and O atoms, respectively. The red-marked adsorption sites stand for the most stable models.

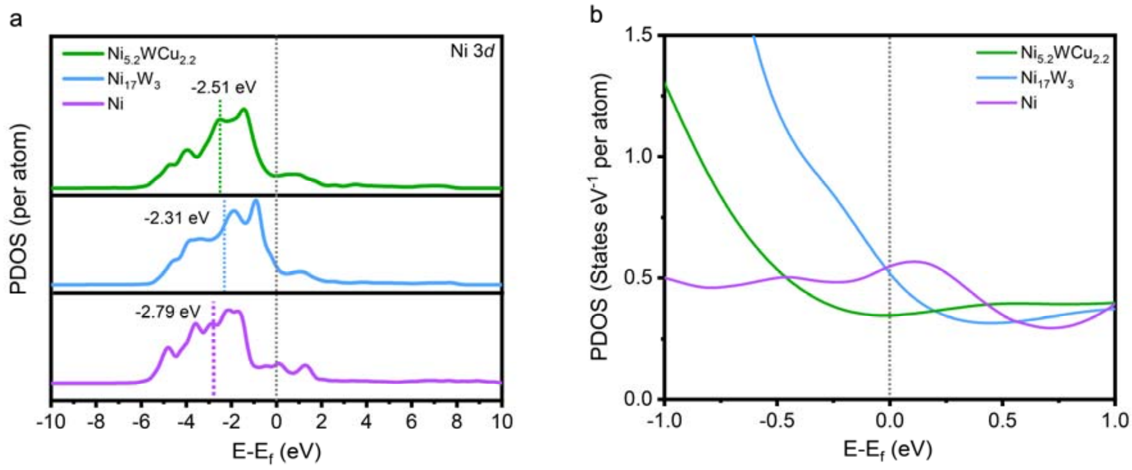

Supplementary Figure 37. a Calculated PDOS of Ni, $Ni_{17}W_3$ and $Ni_{5.2}WCu_{2.2}$ with the Fermi level aligned at 0 eV (gray lines). Purple, blue and green dashed line located at the *d*-band center of Ni, $Ni_{17}W_3$ and $Ni_{5.2}WCu_{2.2}$, respectively. The *d*-band center of Ni atom in single Ni shifts a higher electron density (-2.79 eV) away from the Fermi level, whereas the *d*-band center of Ni atoms relative to the Fermi level are -2.31 eV for $Ni_{17}W_3$ and -2.51 eV for $Ni_{5.2}WCu_{2.2}$ catalyst, indicating that the OH adsorption on catalysts decreases in the order of $Ni_{17}W_3$ > $Ni_{5.2}WCu_{2.2}$ > Ni[8,9]. **b Enlarged PDOS with the Fermi level aligned at 0 eV.** This reveals the higher PODS of single Ni at the Fermi level, which indicates its better charge transfer kinetics and thus a stronger H binding[10]. By contrast, the $Ni_{5.2}WCu_{2.2}$ ternary catalyst exhibits the lowest PDOS at the Fermi level, suggesting a much lower HBE after alloying with W and Cu. The decreased HBE is beneficial to the HOR.

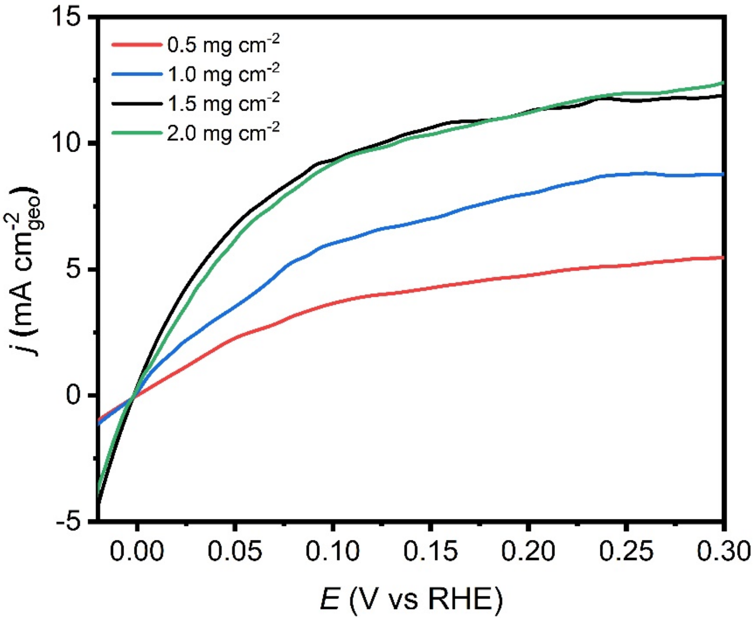

Supplementary Figure 38. HOR polarization curves of Pt/C with different mass loadings on Cu foam. It shows that Pt/C catalyst with a loading of 1.5 mg cm^{-2} exhibits the best HOR performance, in agreement with previous reports on optimized Pt/C loading[11,12].

Supplementary Table 1. EXAFS fitting parameters at the Ni K-edge for the studied samples.

Sample	Shell	N [a]	R (Å) [b]	σ^2 (Å^2·10^{-3}) [c]	ΔE_0 (eV) [d]	R factor (%)
**Ni foil**	Ni-Ni	12	2.48	6.0	6.4	0.1
**Ni**	Ni-Ni	10.8	2.48	5.9	6.4	0.1
**$Ni_{17}W_3$**	Ni-Ni/W	9.0	2.50	6.4	6.9	0.2
**$Ni_{5.2}WCu_{2.2}$**	Ni-Ni/W/Cu	8.7	2.50	6.4	6.6	0.2

[a] N: coordination numbers; [b] R: bond distance; [c] σ^2: Debye-Waller factors; [d] ΔE_0: the inner potential correction. R factor: goodness of fit. S_0^2 were set as 0.80/0.84 for Ni-M (Ni/Cu/W), which was obtained from the experimental EXAFS fit of reference Ni foil reference by fixing CN as the known crystallographic value and was fixed to all the samples.

Supplementary Table 2. Summary of the apparent exchange current density of $Ni_{5.2}WCu_{2.2}$, $Ni_{17}W_3$, Ni, and Pt/C catalyst. The exchange current densities are calculated by micro-polarization methods and Butler-Volmer fitting.

**Catalysts**	**Apparent exchange current density (mA cm^{-2})**	
	**Micro-polarization**	**Butler-Volmer fitting**
**$Ni_{5.2}WCu_{2.2}$**	11.36	11.28
**$Ni_{17}W_3$**	1.23	1.08
**Ni**	0.14	0.12
**Pt/C**	4.99	4.76

Supplementary Table 3. The mass activities and ECSA-normalized j_0 of different catalysts.

Catalysts	Mass activity @ η = 50 mV ($mA\ mg^{-1}_{Ni(Pt)}$)	Mass-normalized j_0 ($mA\ mg^{-1}_{Ni(Pt)}$)	ECSA-normalized j_0 ($mA\ cm^{-2}_{cat.}$)
**$Ni_{5.2}WCu_{2.2}$**	2.55	2.54	0.014
**$Ni_{17}W_3$**	0.93	0.64	0.0071
**Ni**	0.25	0.14	0.0013
**Pt/C**	22.4	16.63	0.0032

Supplementary Table 3. Summary of the DFT-calculated adsorption energy of different models.

**Models**	**DFT-calculated adsorption energy (eV)**			
	**H**	**OH**	**CO**	**O**
**Pt (111)**	-0.45	1.38	-1.78	1.473
**Ni (111)**	-0.58	-0.17		0.133
**$Ni_{17}W_3$ (111)**	-0.51	-0.28	-1.42	0.22
**$Ni_{5.2}WCu_{2.2}$ (111)**	-0.50	-0.21	-1.55	0.31

References

1 Cheon, J. Y. *et al.* Intrinsic Relationship between Enhanced Oxygen Reduction Reaction Activity and Nanoscale Work Function of Doped Carbons. *J. Am. Chem. Soc.* **136**, 8875-8878 (2014).

2 Ni, W. *et al.* Ni_3N as an active hydrogen oxidation reaction catalyst in alkaline medium. *Angew. Chem. Int. Ed.* **58**, 7445-7449 (2019).

3 Duan, Y. *et al.* Bimetallic nickel-molybdenum/tungsten nanoalloys for high-efficiency hydrogen oxidation catalysis in alkaline electrolytes. *Nat. Commun.* **11**, 4789 (2020).

4 Zhang, T. *et al.* Atomically dispersed nickel(I) on an alloy-encapsulated nitrogen-doped carbon nanotube array for high-performance electrochemical CO_2 reduction reaction. *Angew. Chem. Int. Ed.* **59**, 12055-12061 (2020).

5 Yoon, Y., Yan, B. & Surendranath, Y. Suppressing Ion Transfer Enables Versatile Measurements of Electrochemical Surface Area for Intrinsic Activity Comparisons. *J. Am. Chem. Soc.* **140**, 2397-2400 (2018).

6 Xie, X., Chen, S., Ding, W., Nie, Y. & Wei, Z. An extraordinarily stable catalyst: Pt NPs supported on two-dimensional $Ti_3C_2X_2$ (X = OH, F) nanosheets for oxygen reduction reaction. *Chem. Commun.* **49**, 10112-10114 (2013).

7 Tu, W. *et al.* Tungsten as "Adhesive" in $Pt_2CuW_{0.25}$ ternary alloy for highly durable oxygen reduction electrocatalysis. *Adv. Funct. Mater.* **30**, 1908230 (2020).

8 Wang, X. *et al.* Plasma-Triggered Synergy of Exfoliation, Phase Transformation, and Surface Engineering in Cobalt Diselenide for Enhanced Water Oxidation. *Angew. Chem. Int. Ed.* **57**, 16421-16425 (2018).

9 Cai, J. *et al.* N-induced lattice contraction generally boosts the hydrogen evolution catalysis of P-rich metal phosphides. *Sci. Adv.* **6**, eaaw8113 (2020).

10 Yang, J. *et al.* Ultrahigh-current-density niobium disulfide catalysts for hydrogen evolution. *Nat. Mater.* **18**, 1309-1314 (2019).

11 Song, F. *et al.* Interfacing nickel nitride and nickel boosts both electrocatalytic hydrogen evolution and oxidation reactions. *Nat. Commun.* **9**, 4531 (2018).

12 Song, F. *et al.* Interfacial sites between cobalt nitride and cobalt act as bifunctional catalysts for hydrogen electrochemistry. *ACS Energy Lett.* **4**, 1594-1601 (2019).
